# Integration of gene interaction information into a reweighted random survival forest approach for accurate survival prediction and survival biomarker discovery

**DOI:** 10.1038/s41598-018-31497-0

**Published:** 2018-09-04

**Authors:** Wei Wang, Wei Liu

**Affiliations:** 1 Department of Mathematics, Heilongjiang Institute of Technology, Harbin, 150050 China; 2 The Key Laboratory of Molecular Biology for High Cancer Incidence Coastal Chaoshan Area, Shantou University Medical College, Shantou, 515041 China

## Abstract

Accurately predicting patient risk and identifying survival biomarkers are two important tasks in survival analysis. For the emerging high-throughput gene expression data, random survival forest (RSF) is attracting more and more attention as it not only shows excellent performance on survival prediction problems with high-dimensional variables, but also is capable of identifying important variables according to variable importance automatically calculated within the algorithm. However, RSF still suffers from some problems such as limited predictive accuracy on independent datasets and limited biological interpretation of survival biomarkers. In this study, we integrated gene interaction information into a Reweighted RSF model (RRSF) to improve predictive accuracy and identify biologically meaningful survival markers. We applied RRSF to the prediction of patients with glioblastoma multiforme (GBM) and esophageal squamous cell carcinoma (ESCC). With a reconstructed global pathway network and an mRNA-lncRNA co-expression network as the prior gene interaction information, RRSF showed better overall predictive performance than RSF on three GBM and two ESCC datasets. In addition, RRSF identified a two-gene and three-lncRNA signature, which showed robust prognostic values and had high biological relevance to the development of GBM and ESCC, respectively.

## Introduction

Accurately predicting the clinical outcome and response to treatment is a central challenge in clinical cancer research. To achieve precise prognosis, good survival models are needed to assess patient risk, and to identify important predictors that are relevant or predictive of the events such as death or disease recurrence^1,2^.

In survival analysis, the two most widely used survival models are the Cox proportional hazard (Cox PH) model^3^ and random survival forest (RSF)^4^. Due to the emergence of high-throughput gene expression data, RSF is attracting increased attention. It has shown excellent performance on survival prediction problems with high-dimensional variables, and can also cope with complex interaction structures as well as highly correlated variables^5^. Besides predicting patient risk, RSF can rank variables according to their variable importance (VIMP), which reflects the ability to predict outcome and is automatically calculated within the RSF algorithm^4^. These features are considered important advantages given the complexity of high-throughput gene expression data. However, survival prediction models often suffer from several common problems when applied to high-dimensional gene expression data: (i) limited predictive accuracy on independent datasets, (ii) limited reproducibility, and (iii) unclear biological relevance of the predictors used for prediction^6–8^. There are several reasons for this discrepancy, such as genetic heterogeneity across patients, high noise in gene expression measurements, and limited sample size^9^. As the number of genes is much larger than the number of patients, some genes may show a great ability to predict survival outcome purely by chance. Survival prediction models may be impaired if they use such genes as predictors.

To overcome these problems, researchers have proposed to integrate gene interaction information into prediction models. This strategy has successfully improved the predictive performance of both classification^9–11^ and survival prediction models^1,6,7,12–14^. It is based on the hypothesis that topologically important (e.g. highly connected) genes often have important functions in the development of diseases, and tend to show consistent variations in expression across patients^9^. By reweighting the genes according to their topological importance, the predictive model is biased to select topologically important genes as predictors, which could improve the performance of these models in two aspects. Firstly, the robustness of the prediction model may be enhanced, as topologically important genes have consistent variation in expression and low probabilities of correlating with outcomes by chance. Secondly, as the dysregulation of topologically important genes can exert greater influence on a biological system, gene signatures that are identified should have a higher biological relevance with the given disease. Recently, researchers have focused on identifying long non-coding RNA (lncRNA) signatures for survival prediction^15,16^. As the functions of lncRNAs are largely unknown, integrating gene interaction information to identify biologically meaningful lncRNA signatures will be an effective way in this context. Gene interaction information has successfully been integrated into the Cox PH model, such as Net-Cox^1^ and PathBoost^14^. However, whether the performance of RSF could be improved following the integration of interaction information has not been investigated.

On the other hand, RSF often needs a relatively large number of features to achieve accurate prediction. While, for clinical utilization, biomarkers with a limited number of genes are urgently required. Thus, we propose a novel pipeline that integrates gene interaction information into a Reweighted RSF (RRSF) approach to improve predictive performance and select important genes associated with survival. Then we use the Cox PH model to identify robust biomarkers from these genes based on an exhaustive search. We applied RRSF to patients with glioblastoma multiforme (GBM) and esophageal squamous cell carcinoma (ESCC) to evaluate its predictive performance and identify biomarkers.

## Materials and Methods

### Datasets

Three GBM and two ESCC datasets were collected for evaluating RRSF. The first GBM dataset (referred to as “GBM-TCGA”) was obtained from The Cancer Genome Atlas (TCGA)^17^. We downloaded the processed, level 3, gene expression and associated clinical data. Samples with Karnofsky’s score of <70, which is indictive of patients who might have died for reasons other than the disease itself^18^ were eliminated. We mapped the probes to the Entrez gene ID and, by averaging their expression values, merged those that mapped to the same Entrez ID. This resulted in a total of 15,686 profiled genes from 314 patients. The other two independent GBM datasets (GSE4412^19^ and GSE4271^20,21^) were downloaded from the Gene Expression Omnibus database^22^. We eliminated samples for which survival data was unavailable and also mapped the probes to Entrez gene ID. This resulted 13,434 profiled genes from 85 and 77 samples for the GSE4412 and GSE4271 datasets, respectively.

The two ESCC datasets, comprising 119 (GSE53624) and 60 (GSE53622) patients, respectively, were downloaded from the GEO database. Both datasets were profiled using the Agilent-038314 CBC Homo sapiens lncRNA + mRNA microarray V2.0 platform. By using nucleotide sequences similarity search tool BLASTn^23^, the Agilent human lncRNA + mRNA microarray probe sets were reannotated by mapping all probes to the lncRNA and mRNA transcripts obtained from the GENCODE database (GRCh38, release 21)^24^. Probes that mapped uniquely to the lncRNA or mRNA transcripts with no mismatches were kept, thereby resulting in the inclusion of 17,434 mRNAs and 6,252 lncRNAs.

### Methods

#### Construction of global pathway network

The global pathway network was constructed on interaction data from the Kyoto Encyclopedia of Genes and Genomes database (KEGG)^25^ by using the iSubpathwayMiner^26^ R package. First, each of the 343 KEGG pathways were converted into a directed graph. In each graph, each node represented a gene or a metabolite. The directions of edges were determined based on the biochemical reaction information contained in KGML files available in KEGG. For example, in a signaling pathway, if gene A activates (or inhibits) gene B, then the direction is “A → B”. In a metabolic pathway, the direction is “substrate ↔ enzyme ↔ product” for reversible reactions and “substrate → enzyme → product” for irreversible reactions. As directed graphs may contribute towards a more accurate evaluation of the topological importance of genes^9^, these were preferentially constructed over undirected graphs. The resulting 343 graphs were then merged into a global pathway network, where those genes that are located in multiple pathways merging into one node. The final global pathway network contained 7159 nodes and 39930 edges.

#### Construction of the co-expression network

The co-expression network was constructed by using the WGCNA^27^ R package. Pearson correlation coefficients and corresponding Student *P*-values were calculated between all the gene pairs. Next, following Benjamini and Hochberg correction, gene pairs with a *P*-value < 1 × 10^−7^ (Benjamini and Hochberg correction) were used to construct the co-expression network. For GBM, a gene-gene co-expression network was constructed based on the GBM-TCGA dataset and contained 4,714 genes and 823,942 edges. For ESCC, an mRNA–lncRNA co-expression network was constructed based on ESCC-train. The mRNA–lncRNA co-expression network contained 13,445 nodes (10,069 mRNAs and 3,376 lncRNAs) and 1,426,156 edges.

#### Evaluation of topological importance

The directed random walk (DRW) algorithm^9^ was used to evaluate the topological importance of genes in the gene interaction network. The DRW algorithm simulates a random walker that starts at a source node and transits from its current node to a randomly chosen neighboring node or goes back to the source node with a restart probability of *r* at each time step. After a finite number of steps, the probability distribution of the random walker being at each node in the gene interaction network will reach a steady state. This probability distribution reflects the topological importance of nodes (genes) in the gene interaction network. Let **M** be the row-normalized adjacency matrix of the gene interaction network. The DRW algorithm is thus formally defined as:

1 $${{\bf{W}}}_{t+1}=(1-r){{\bf{M}}}^{T}{{\bf{W}}}_{t}+r{{\bf{W}}}_{0}$$

where **W**_*t*_ is a weight vector in which the *i*th element holds the probability of being at node *i* at time step *t*, **W**_0_ is the initial weight vector (initial probability distribution) at time step *t* = 0, while *r* is the restart probability ranging from 0 to 1. We constructed **W**_0_ by assigning −log(*P*_*i*_) as its *i*th element and normalized it to a unit vector, where *P*_*i*_ was the Cox *P*-value of node (gene) *i*. The restart probability *r* was set as 0.3. By iterating formula (1), **W**_*t*_ was recursively updated according to the topological structure of the gene interaction network (represented by adjacency matrix **M** in formula (1)), and converged to a steady state **W**_∞_ when the *L*_1_-norm between **W**_*t*_ and **W**_*t*+1_ was less than 10^−10^. **W**_∞_ is the probability distribution at the steady state, which provides a measure of the topological importance of the genes in the gene interaction network. We refer to **W**_∞_ as the topological weights. Its *i*th element holds the topological weight of gene *i*. Genes that i) have large degrees in the gene interaction network; ii) have significant *P*-values; and iii) are close to a large number of genes that also have large topological weights will obtain larger topological weights.

#### RRSF model construction

RSF^4^ is a non-parametric ensemble tree learning method for survival outcome prediction. An RSF ensemble comprises a collection of recursively partitioned binary trees with random components. Each tree is constructed on an independent bootstrap sample and grown deeply. During the growing process, each node is split using a survival splitting rule based on a randomly selected subset of genes. In general, RSF treats genes equally and randomly selects genes according to a uniform probability distribution.

RRSF is similar to RSF but integrates the topological importance of genes. Considering that the topological importance of genes is different, RRSF selects genes for node splitting according to their topological weight. Genes with larger topological weights will be selected with larger probabilities. The RRSF model is constructed using the following four steps:

1. Draw *ntree* bootstrap samples from the training set. In our examples, *ntree* = 1000 was used.
2. Grow a survival tree for each bootstrap sample. During the growing process, *mtry* genes are randomly selected as candidate genes for each node splitting according to their topological weights. We used the default setting *mtry* = sqrt(*p*), where *p* is the number of genes. The node is split using the gene that maximizes the difference in survival between child nodes. The difference in survival is evaluated by a log-rank splitting rule.
3. Grow each tree to full size under the constraint that each leaf node contains no less than *nodesize* deaths. (We used *nodesize* = 3 in our example.)
4. Calculate the cumulative hazard function (CHF) using the Nelson-Aalen estimator^4^ for each leaf node in each tree. Samples in the same leaf node have the same CHF. The *ntree* fitted survival trees constitute the RRSF ensemble.

To predict the risk of a new sample, we drop the sample down the *ntree* survival trees, respectively. For each tree, the sample will fall into a unique leaf node. Thus, the sample will fall into a total of *ntree* leaf nodes. Next, the averaged CHF of the *ntree* leaf nodes is taken as the CHF of the new sample. The predicted value of the new sample is defined as the sum of its CHF over the event times. RRSF was implemented based on the randomForestSRC^4^ R package.

#### Selection process for genes with best predictive performance

We used Breiman–Cutler permutation VIMP described by Breiman^28^ to evaluate the importance of genes. The 10% lowest ranking genes were discarded at each level. The remaining 90% of genes were used as a new feature set to construct RRSF models at next level. This process was iterated until only two genes were left in the feature set.

#### Identifying biomarkers based on exhaustive searches

Starting from the 10 genes identified by RRSF, Cox PH models were constructed on a training set using all combinations of the 10 genes. This resulted in the construction of 2^10^-1 = 1023 Cox PH models. These models were used to predict the risk of patients in a test set. C-indices were then calculated to evaluate the predictive performance of the gene combinations. The final biomarkers were selected by considering the balance between C-index and the number of genes involved.

#### Model evaluation

The predictive performance of the RRSF model was assessed using the C-index^29^, which measures the proportion of all usable patient pairs for which the predicted values and actual survival times are concordant. The C-index was calculated using the “survcomp”^30,31^ R package.

#### Comparison experiments for RRSF and RSF

**Figure 1:**
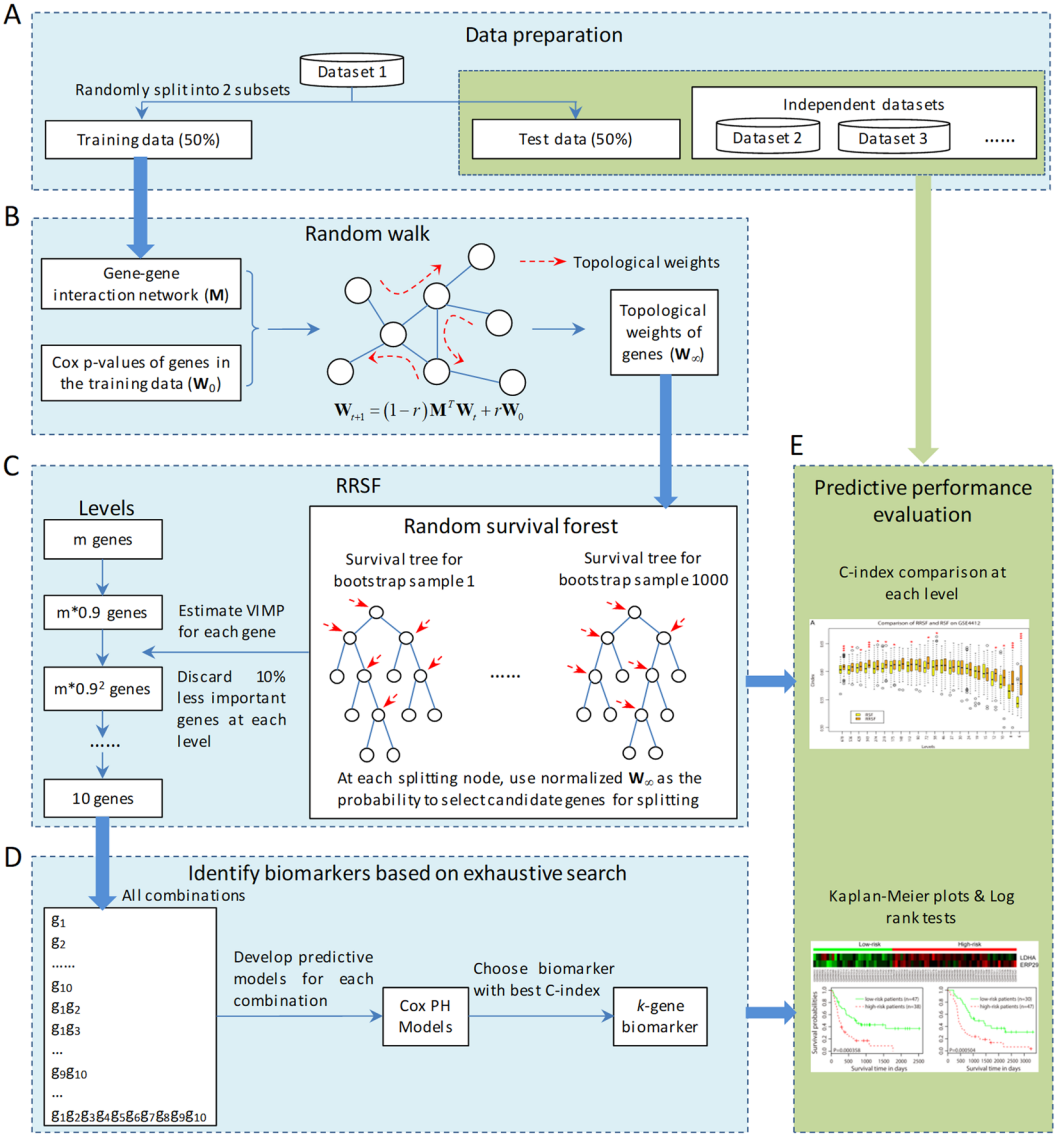
The pipeline to evaluate the predictive performance of RRSF. (**A**) Data preparation. Dataset 1 was randomly split into a training set (50%) and a test set (50%). The training set was used to train the RRSF model, while the test set and independent datasets were used to evaluate its predictive performance. (**B**) Topological weights of genes were inferred using DRW. (**C**) Selection process for genes with best predictive performance. The number of genes were narrowed down by several iterative steps, in which, according to their VIMPs, genes ranking in the lowest 10% in terms of importance were discarded at each step. (**D**) Development of predictive models for all combinations of the 10 genes. Biomarkers with the highest C-index were identified. (**E**) Evaluation of predictive performance by C-index, Kaplan–Meier curves, and log-rank tests.

For both GBM and ESCC, we prepared multiple datasets to evaluate the performance of RRSF (Fig. 1A). The training set was used for univariate Cox regression (Fig. 1B), RRSF model construction and gene selection (Fig. 1C), and Cox PH model construction and biomarker identification based on an exhaustive search (Fig. 1D). The test set and independent datasets were used to evaluate the predictive performance of RRSF and identified biomarkers (Fig. 1E). The RSF was evaluated in the same way. For fair comparison, at each level, 100 models were constructed and the overall predictive performance compared using the C-index. Furthermore, to investigate the patient stratification ability of the two models, the two methods were compared using Kaplan–Meier survival curves and the log-rank test.

#### Enrichment analysis

The GO functional enrichment was performed using DAVID^32,33^. Enriched P values were calculated by hypergeometric test, and then subjected to Benjamini and Hochberg correction.

## Results

To evaluate the proposed method, the RRSF model was applied to the survival prediction of two cancers, GBM and ESCC, respectively. For each cancer type, a pipeline was designed to evaluate the predictive performance. This was achieved by following five steps, which included: i) data preparation; ii) evaluation of the topological weights of the genes using the DRW algorithm; iii) model evaluation of RRSF and selection process for genes with the best predictive performance; (iv) biomarker discovery based on exhaustive searches; and (v) predictive performance evaluation using Harrell’s concordance index (C-index), Kaplan–Meier curves, and log-rank tests (Fig. 1).

## GBM

Three independent datasets (GBM-TCGA, GSE4412, and GSE4271) were collected for GBM patients (see Materials and Methods). The GBM-TCGA dataset was randomly split into a training set (GBM-TCGA-train, *n* = 157) and a test set (GBM-TCGA-test, *n* = 157). The clinicopathological characteristics were comparable in these two sets (Table 1). GBM-TCGA-train was used to train the RRSF model. GBM-TCGA-test, GSE4412, and GSE4271 were used to evaluate the predictive performance of the RRSF model.

**Table 1:** Clinical characteristics of patients in GBM datasets.

Characteristic	GBM-TCGA-train	GBM-TCGA-test	*P* ^a^	GSE4412	*P*	GSE4271	*P*
NO. of samples	157	157		85		77	
Age (median)	54	59	0.3584*	42	4.98 × 10^−07^*	45	2.03 × 10^−06^*
Gender			0.9072		0.0005		0.4749
Male	97	99		32		52	
Female	60	58		53		25	
Death at follow-up			0.6059		0.3563		0.5190
Yes	119	114		59		62	
No	38	43		26		15	
Median survival (days)	385	394	0.8346*	389	0.5901*	665	2.94 × 10^−5^*
Platform	G4502A_07^b^	G4502A_07		GPL96^c^		GPL96	
NO. of genes	15686	15686		13434		13434	

^a^*P*-values were calculated by the Fisher’s exact test, unless otherwise stated. ^b^G4502A_07: Agilent 244 K Custom Gene Expression G4502A−07. ^c^GPL96: Affymetrix Human Genome U133A Array. *Wilcoxon rank sum test.

To evaluate topological importance, DRW was performed on a reconstructed global pathway network (see Materials and Methods). First, by mapping 15686 genes in the GBM-TCGA-train dataset to the global pathway network, 4853 common genes were obtained. The *P*-values from the univariate Cox regression analysis of these genes were used to initialize DRW and evaluate the topological weights (Fig. 1B). The topological weights of genes that were significant at the 0.05 level following univariate Cox regression analysis were listed in Table S1. We ranked the genes according to their topological weights. Genes that had a large degree or a small *P*-value tended to obtain a large topological weight (Fig. S1), indicating that the topological weights could reflect the topological importance of genes.

### RRSF predicted survival outcomes of GBM patients with higher accuracy than RSF

To train RRSF models, 670 genes were selected as the initial feature set. These genes were common in four GBM datasets as well as the global pathway network, and had significant Cox *P*-values (<0.05) in GBM-TCGA-train. The RRSF models were trained based on the 670 genes from GBM-TCGA-train, and tested on the remaining three GBM datasets. At each level, the VIMP for each gene was estimated and those that ranked in the lowest 10% in terms of importance were discarded. The remaining 90% of genes were used as a new feature set for next RRSF model construction (Fig. 1C). This process was repeated until two genes were left in the feature set. For an unbiased evaluation, 100 RRSF models were constructed at each level, using the average C-index to evaluate the overall performance. The RSF model was evaluated following the same procedure.

**Figure 2:**
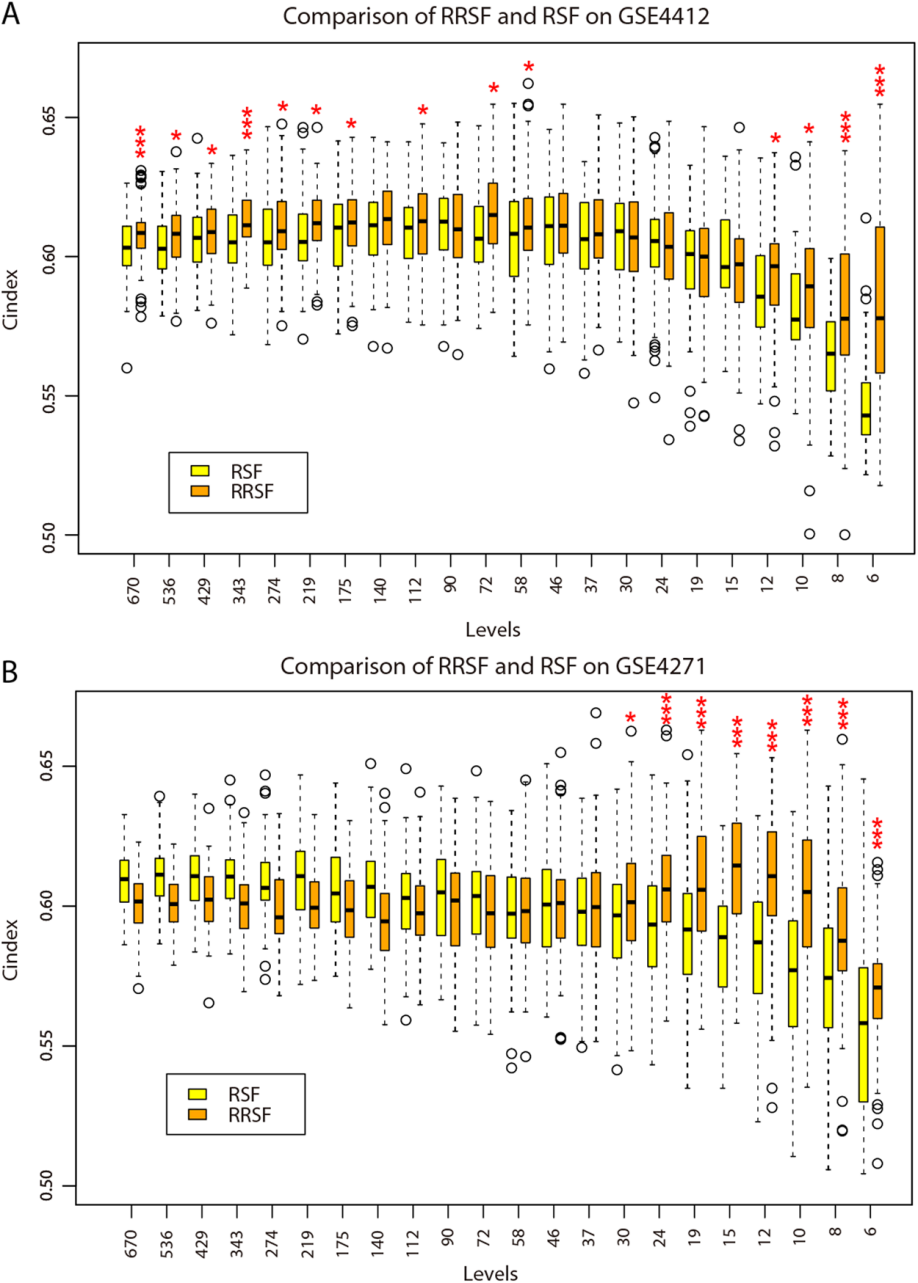
Performance comparison of RRSF and RSF models on the GBM datasets. (**A**) Boxplots of 500 C-indices on the GSE4412 dataset at each level by RRSF and RSF modelling (Wilcoxon signed-rank test, *P < 0.05, ***P < 0.001). (**B**) Boxplots of 500 C-indices on the GSE4271 dataset at each level by RRSF and RSF modelling.

Both RRSF and RSF obtained small training errors when the number of features included in the models ranged from 6 to 30 (Figs S2 and S3). Too many or too few features did not perform well. Although RSF performed better on the training set, its predictive performance declined dramatically on not only the GBM-TCGA-test, but especially on the GSE4412 and GSE4271 independent datasets. The mean C-index was larger for RRSF than RSF at all levels with less than 50 features (Fig. 2). The C-indices were significantly larger for RRSF than RSF at levels 6–12 on the GSE4412 (median C-index: 0.5779 *vs* 0.5429, Wilcoxon signed rank test, *P* = 6.02 × 10^−15^; 0.5778 *vs* 0.5651, *P* = 2.70 × 10^−6^; 0.5893 *vs* 0.5774, *P* = 8.59 × 10^−3^; and 0.5966 *vs* 0.5856, *P* = 1.74 × 10^−2^, respectively), and the GSE4271 (median C-index: 0.5709 *vs* 0.5582, Wilcoxon signed rank test, *P* = 4.73 × 10^−4^; 0.5876 *vs* 0.5744, *P* = 2.76 × 10^−6^; 0.6051 *vs* 0.5771, *P* = 6.17 × 10^−11^; and 0.6107 *vs* 0.5871, *P* = 1.08 × 10^−10^, respectively). Despite C-index comparisons, the ability of the two models to stratify GBM patients was also investigated. GBM patients were stratified into a high-risk and a low-risk group using the mean of the predicted values calculated by RRSF and RSF models at the 10-gene level. Both RRSF and RSF estimated CHF had low survival probabilities for patients in the high-risk group and high survival probabilities for patients in the low-risk group (Fig. S4). The difference in survival curves based on forest estimated CHF were not obvious. However, patients in the high-risk group stratified by RRSF showed more consistent low survival probabilities in the GBM-TCGA-test (Fig. S4B vs S4F), GSE4412 (Fig. S4C vs S4G) and GSE4271 datasets (Fig. S4D vs S4H). Kaplan–Meier survival curves based on the raw survival data were further investigated (Fig. S5). Both RRSF and RSF stratified GBM patients in GBM-TCGA-train into two groups with significant survival differences (log-rank test, *P* < 0.0001), but RRSF gave better stratifications on the remaining three datasets (RRSF vs RSF, log-rank test, GBM-TCGA-test: *P* = 0.03 vs *P* = 0.62; GSE4412: *P* < 0.0001 vs *P* = 0.0033; and GSE4271: *P* = 0.049 vs *P* = 0.77, Fig. S5). The smaller the log-rank *P* value, the better the stratification. It indicates that RRSF could yield better overall performance by integrating gene interaction information.

To assess the risk of false positive findings using RRSF, the survival outcome of patients in the GBM-TCGA-train dataset were permuted and the predictive performance of RRSF models evaluated as earlier. Results showed that, like RSF, RRSF obtained C-indices ~ 0.5 at almost all levels on each of the three test datasets (Fig. S6), which were not better than random. This confirms the credibility of the prediction results of RRSF on GBM datasets.

### A two-gene signature predicts survival outcome of patients with GBM

To identify biomarkers for GBM, the lowest number of genes that can predict survival outcomes accurately need to be selected. So we started from the 10 genes identified by RRSF at the 10-gene level, where RRSF obtained a favorable predictive performance (Fig. 2). The topological weights and degrees of the top 10 most frequently selected genes in 100 repeated models were larger for RRSF (Table 2) than RSF (Table S2). This indicated that topological weights contributed to the identification of topologically important genes using the RRSF method. Among these 10 genes, five have been shown to play key roles in regulating GBM cell proliferation, invasion, and apoptosis and were suggested as potential therapeutic targets. These included *HDAC2*, *OSMR*, *LDHA*, *SPP1*, and *RAP1A* (Table 2). As RRSF (RSF) models with too few genes did not yield good performance (Figs S2 and S3), we resorted to Cox PH models combined with an exhaustive search (Fig. 1D). A total of 1023 Cox PH models were constructed for all combinations of the 10 genes. By considering a balance between C-index outcomes and the number of genes, a two-gene signature, which included *LDHA* and *ERP29*, was identified (Fig. S7).

**Table 2:** Ten genes identified by RRSF at 10-gene level.

Entrez Gene ID	Gene Symbol	Cox P-value	Degree	Topological weight	Description	Reference (PMID)
3066	*HDAC2*	1.26 × 10^−02^	100	5.74 × 10^−04^	Silencing of *HDAC2* suppresses malignancy for proliferation, migration, and invasion of glioblastoma cells.	27832326
838	*CASP5*	5.65 × 10^−04^	3	5.11 × 10^−04^	—	—
9180	*OSMR*	6.15 × 10^−04^	41	4.17 × 10^−04^	*OSMR* plays a key role in driving the growth of deadly GBM tumours, and can be explored as potential target for therapeutic intervention.	27110918, 25748242
3939	*LDHA*	1.12 × 10^−04^	9	3.96 × 10^−04^	Silencing of *LDHA* inhibits glycolysis, cell proliferation, invasion, and promotes cell apoptosis by downregulation of the Warburg effect in GBM.	26694942, 26494310, 26269128
6696	*SPP1*	1.85 × 10^−04^	22	3.42 × 10^−04^	High expression of *SPP1* is associated with poor survival outcome in human GBM.	25658639, 25961929
10961	*ERP29*	2.42 × 10^−04^	2	3.37 × 10^−04^	—	—
5906	*RAP1A*	1.53 × 10^−03^	25	3.35 × 10^−04^	*RAP1A* mediates thrombin-stimulated, integrin-dependent GBM cell proliferation and tumor growth.	24790104
10552	*ARPC1A*	9.44 × 10^−03^	12	3.35 × 10^−04^	—	—
79695	*GALNT12*	4.65 × 10^−03^	1	3.05 × 10^−04^	—	—
55341	*LSG1*	1.99 × 10^−02^	4	2.45 × 10^−04^	—	—

**Figure 3:**
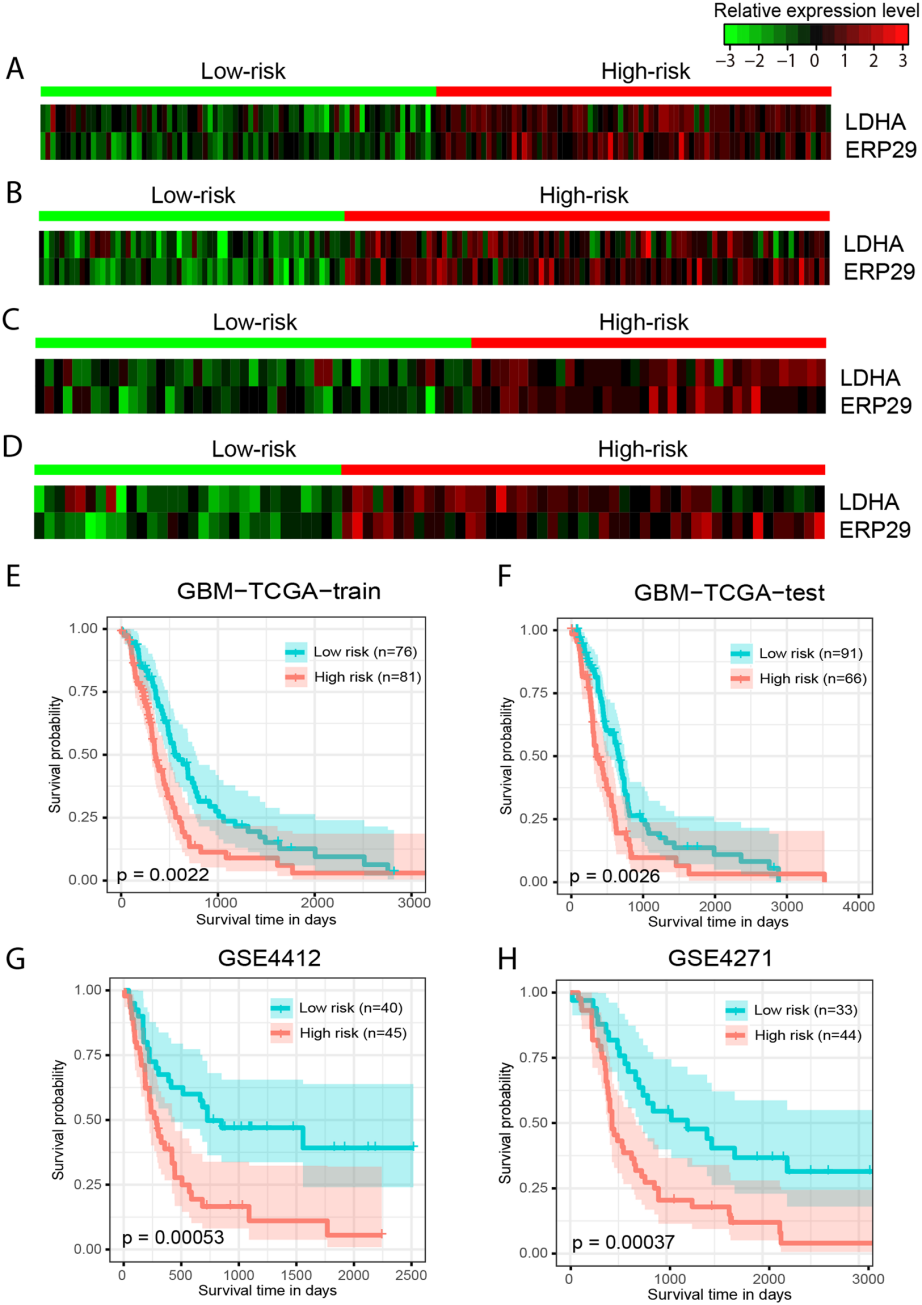
The two-gene signature predicts overall survival of patients with GBM. Heat maps of z-score transformed expression values for each gene (**A**–**D**), and Kaplan–Meier survival curves of patients classified into high- and low-risk groups using the two-gene signature (**E**–**H**). P values were calculated using the log-rank test. (**A**,**E**) GBM-TCGA-train dataset, 157 patients. (**B**,**F**) GBM-TCGA-test dataset, 157 patients. (**C**,**G**) GSE4412 dataset, 85 patients. (**E**,**H**) GSE4712 dataset, 77 patients.

The two-gene signature Cox PH model obtained a C-index of 0.6342 on the GBM-TCGA-train dataset. Using the mean of the predictive values as the cutoff, patients in this dataset were stratified into a high-risk (*n* = 81) and a low-risk group (*n* = 76). Both *LDHA* and *ERP29* were highly expressed in the high-risk group (Fig. 3A). Patients in the high-risk group had significantly shorter overall survival than those in the low-risk group (median survival 298.5 days *vs* 473 days, *P* = 0.0022, Fig. 3E). The two-gene signature was then tested for its prognostic value in the GBM-TCGA-test, GSE4412 and GSE4271 datasets. These obtained C-indices of 0.6360, 0.6078, and 0.6105, respectively. Using the same cutoff value as that used in GBM-TCGA-train dataset, patients in the three subsequent datasets were stratified into a high-risk and a low-risk group. As in GBM-TCGA-train dataset, expression of both *LDHA* and *ERP29* were consistently higher in the high-risk group than in the low-risk group (Fig. 3B–D). Respectively, patients in the high-risk group had significantly shorter overall survival than those in the low-risk group for the GBM-TCGA-test (median survival 315 days *vs* 463 days, *P* = 0.0026, Fig. 3F), GSE4412 (median survival 265 days *vs* 726 days, *P* = 5.32 × 10^−4^, Fig. 3G), and GSE4271 datasets (median survival 434 days *vs* 1022 days, *P* = 3.66 × 10^−4^, Fig. 3H). This indicates that the two-gene signature has robust prognostic value in independent datasets.

## ESCC

Next, the RRSF model was applied to the survival prediction of ESCC patients. This was done to investigate whether the RRSF method could obtain better outcomes based on lncRNA expression data. Two independent ESCC datasets (GSE53624 and GSE53622), which collectively contained 6252 lncRNAs, were obtained (see Materials and Methods). The GSE53624 dataset was randomly split into a training set (ESCC-train, 60 samples) and a test set (ESCC-test, 59 samples). The clinicopathological characteristics were comparable in these two sets (Table 3). The ESCC-train dataset was used to train the RRSF model, while the ESCC-test and the independent GSE53622 (ESCC-valid) datasets were used to evaluate the predictive performance of the RRSF model.

**Table 3:** Clinical characteristics of patients in ESCC datasets.

Characteristic	ESCC-train	ESCC-test	*P*	ESCC-valid	*P*
NO. of samples	60	59		60	
Age (median)	59	59	0.8564*	60.23973	0.6180*
Gender			0.6610		1
Male	48	50		48	
Female	12	9		12	
Smoke			0.1340		0.8531
Yes	36	44		34	
No	24	15		26	
Alcohol			0.7591		0.5805
Yes	36	38		32	
No	24	21		28	
Tumour location			0.4270		0.1031
Upper	7	7		6	
Middle	38	31		28	
Lower	15	21		26	
Tumour grade			0.1966		0.2891
Well differntiated	16	8		9	
Moderately differentiated	29	35		34	
Poorly differentiated	15	16		17	
T stage			0.6019		0.0002
T1	3	5		4	
T2	12	8		7	
T3	29	33		48	
T4	16	13		1	
N stage			0.1938		0.6360
N0	24	30		29	
N1	26	16		20	
N2	7	6		9	
N3	3	7		2	
TNM stage			0.2333		0.1900
I	1	5		4	
II	25	22		30	
III	34	32		26	
Death at follow-up			1		0.5786
Yes	37	36		33	
No	23	23		27	
Median survival (months)	31.9833	32.2	0.9576*	39.3333	0.1825*

P-Values are calculated by χ2 test or Fisher’s exact test, unless otherwise stated. *Wilcoxon rank-sum test.

### RRSF predicted survival outcomes of ESCC patients with higher accuracy than RSF

**Figure 4:**
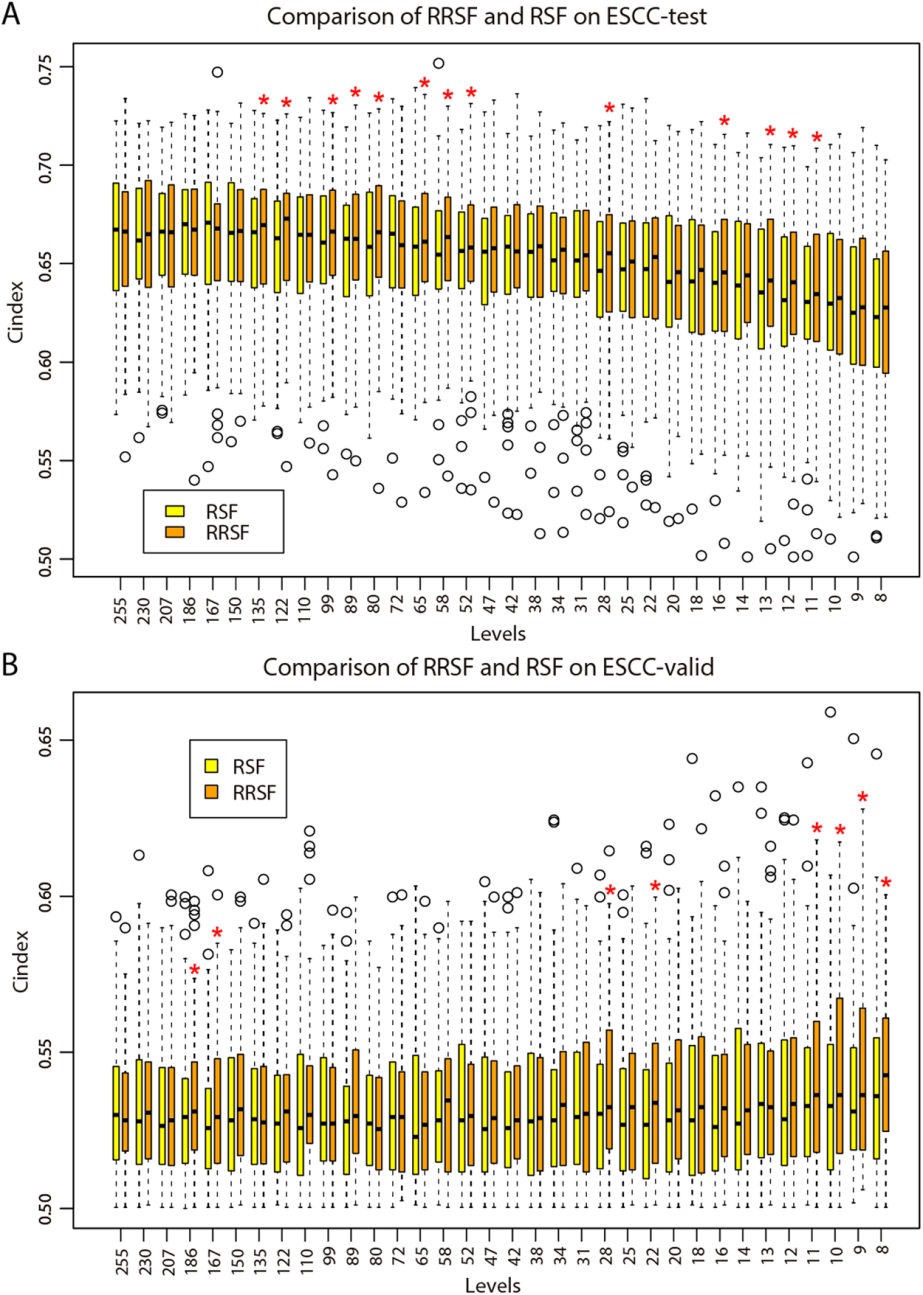
Performance comparison of RRSF and RSF models on the ESCC datasets. (**A**) Boxplots of 500 C-indices on the ESCC-test dataset at each level by RRSF and RSF modelling (Wilcoxon signed-rank test, *P < 0.05, ***P < 0.001). (**B**) Boxplots of 500 C-indices on the ESCC-valid dataset at each level by RRSF and RSF modelling.

The topological weights of lncRNAs were evaluated from an mRNA–lncRNA co-expression network using DRW (Table S3). As with the genes in the GBM datasets, lncRNAs that had a large node degree or small *P*-value tended to obtain greater topological weight (Fig. S8). The topological weights of lncRNAs were integrated to train RRSF models based on the ESCC-train dataset. Using the 255 lncRNAs that had a significant Cox *P*-value (<0.05) as the initial feature set, RRSF and RSF models were constructed at different levels following the same procedures described for the GBM datasets (Fig. 1C). The RRSF models obtained larger C-indices than RSF at almost all levels on both the ESCC-test and ESCC-valid datasets (Fig. 4). This advantage became more obvious when the number of lncRNAs decreased. For example, respectively, C-indices were significantly larger for RRSF than RSF models on ESCC-test data at levels 13 (median C-index: 0.6456 *vs* 0.6403, *P* = 0.034), and 12 (0.6405 *vs* 0.6314, *P* = 0.045), and on ESCC-valid data at levels 10 (0.5422 *vs* 0.5368, *P* = 0.036), nine (0.5433 *vs* 0.5365, *P* = 0.016), and eight (0.5441 *vs* 0.5385, *P* = 0.025). The ability of the two models to stratify ESCC patients was also investigated. ESCC patients were stratified into a high-risk and a low-risk group using the mean of the predicted values calculated by RRSF and RSF models at the 10-gene level. Both RRSF and RSF estimated CHF showed low survival probabilities for patients in the high-risk group and high survival probabilities for patients in the low-risk group (Fig. S9). Kaplan–Meier survival curve analysis based on the raw survival data confirmed the advantage of RRSF over RSF models (Fig. S10). Both RRSF and RSF stratified ESCC patients in the ESCC-train dataset into two groups with significant differences in survival (log-rank test, *P* < 0.0001). However, when compared to RSF, RRSF gave better stratification outcomes on the two test datasets (log-rank test, ESCC-test: *P* = 0.0014 vs *P* = 0.016; and ESCC-valid: *P* = 0.1 vs *P* = 0.55). This indicates that, by integrating mRNA–lncRNA co-expression information, RRSF could yield better predictive performance.

The risk of false positive findings when applying RRSF models to ESCC datasets was assessed by survival outcome permutation of patient data contained in the ESCC-train set. Trained on the ESCC-train dataset with permuted patient survival outcomes, RRSF models obtained C-indices around 0.5 on almost all levels on the two test datasets (Fig. S11), which were not better than random. This confirms the credibility of the prediction results of the RRSF method applied to the ESCC datasets.

### A three-lncRNA signature predicts survival outcome of ESCC patients

To identify biomarkers for ESCC, we also started from the 10 lncRNAs identified by RRSF at the 10-gene level, where RRSF obtained a favorable predictive performance (Fig. 4). As with GBM, the topological weights and degrees of the top 10 most frequently selected lncRNAs in 100 models were larger for RRSF (Table S4) when compared to RSF models (Table S5), further indicating the effect of integrating topological weights. By performing an exhaustive search on 1023 Cox PH models for all combinations of the 10 lncRNAs (Table S4), and by considering a balance between the C-index value and the number of lncRNAs included, a three-lncRNA signature, which included *MAMDC2-AS1*, *AC146944*.*4*, and *AP003108*.*1*, was identified (Fig. S12).

**Figure 5:**
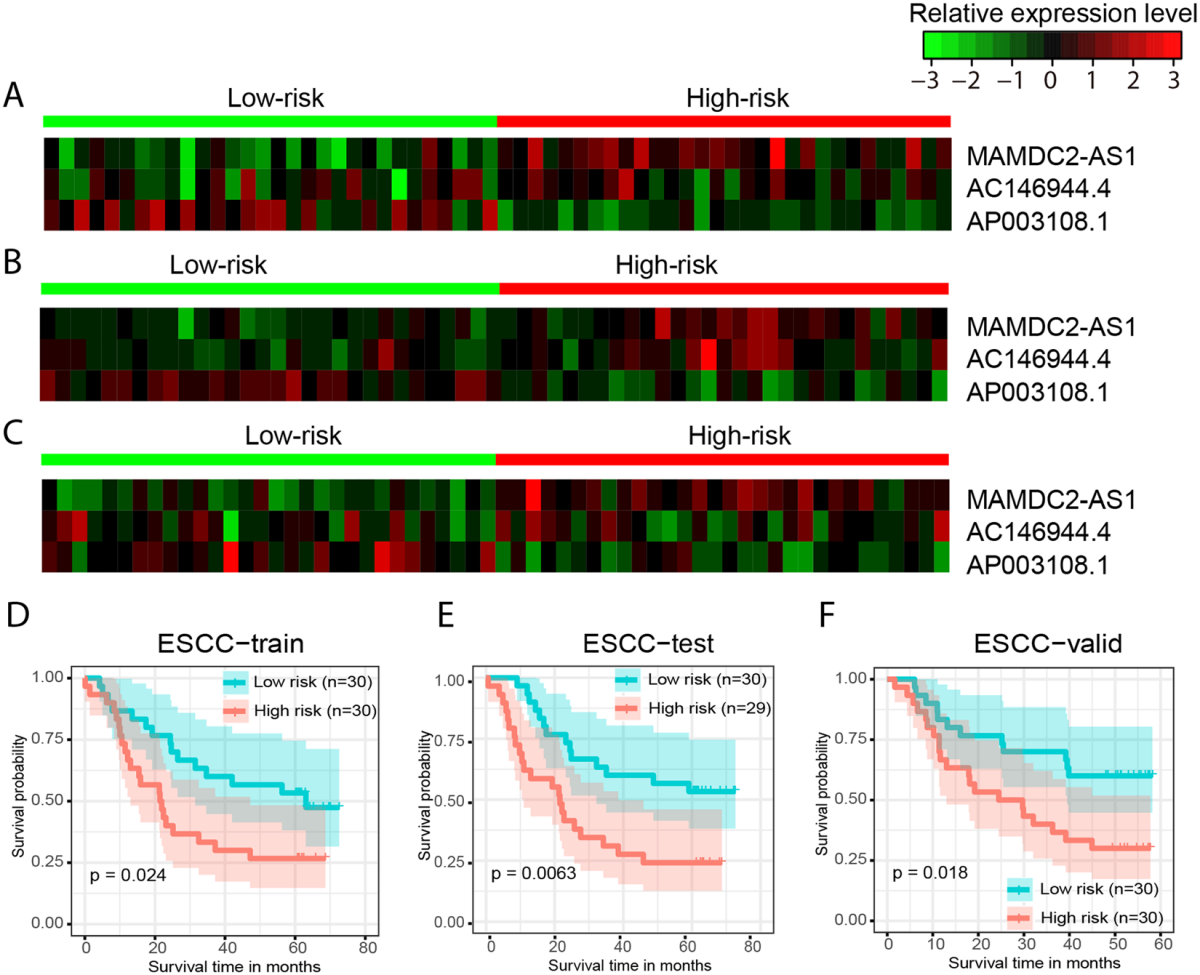
The three-lncRNA signature predicts overall survival of patients with ESCC. Heat maps of z-score transformed expression values for each lncRNA (**A**–**C**), and Kaplan–Meier survival curves of patients classified into high- and low-risk groups using the three-lncRNA signature (**D**–**F**). P values were calculated using the log-rank test. (**A**,**D**) ESCC-train dataset, 60 patients. (**B**,**E**) ESCC-test dataset, 59 patients. (**C**,**F**) ESCC-valid dataset, 60 patients.

With the three-lncRNA signature, patients in the ESCC-train dataset were stratified into a high-risk group (*n* = 30) and a low-risk group (*n* = 30) using the mean of the predictive values as the cutoff. *MAMDC2-AS1* was highly expressed in the high-risk group, while *AP003108*.*1* was highly expressed in the low-risk group (Fig. 5A). Patients in the high-risk group had significantly shorter overall survival than those in the low-risk group (median survival 21.68 months *vs* 60.35 months, *P* = 0.0241, Fig. 5D). The three-lncRNA signature was then tested for its prognostic value in the ESCC-test and ESCC-valid datasets. These obtained C-indices of 0.7202 and 0.5526, respectively. Using the same cutoff as that used in ESCC-train set, patients in these two datasets were stratified into a high-risk group and a low-risk group. All the three lncRNAs exhibited similar expression patterns in the ESCC-test and ESCC-valid datasets as that observed in the ESCC-train dataset (Fig. 5B–C). Patients in the high-risk group had significantly shorter overall survival than those in the low-risk group for ESCC-test (median survival 21.53 months *vs* 60.43 months, *P* = 0.0063, Fig. 5E) and ESCC-valid (median survival 27.08 months *vs* 49.3 months, *P* = 0.0178, Fig. 5F), respectively. This indicated that RRSF could identify robust lncRNA signatures. In addition, the three-lncRNA signature gave a slightly better stratification on ESCC-test (log-rank P = 0.0063 vs 0.031) and ESCC-valid (log-rank P = 0.0178 vs 0.049) than N-stage (Fig. S13), a known predominant prognostic factor for ESCC^34^. Multivariable analysis showed that the three-lncRNA signature and N-stage were independent prognostic factors for ESCC patients in both GSE53624 (the three-lncRNA signature: hazard ratio (HR) = 2.2438, 95% confidence interval (CI) 1.3320 to 3.78, *P* = 0.0024; N-stage: HR = 1.9975, 95%CI 1.1978 to 3.3310, *P* = 0.0080) and the independent cohort GSE53622 (the three-lncRNA signature: HR = 2.8782, 95%CI 1.3308 to 6.2249, *P* = 0.0072; N-stage: HR = 3.0147, 95% CI 1.3271 to 6.8480, *P* = 0.0084) (Table S6).

### LncRNAs selected by RRSF provide biologically informative models for ESCC development

**Figure 6:**
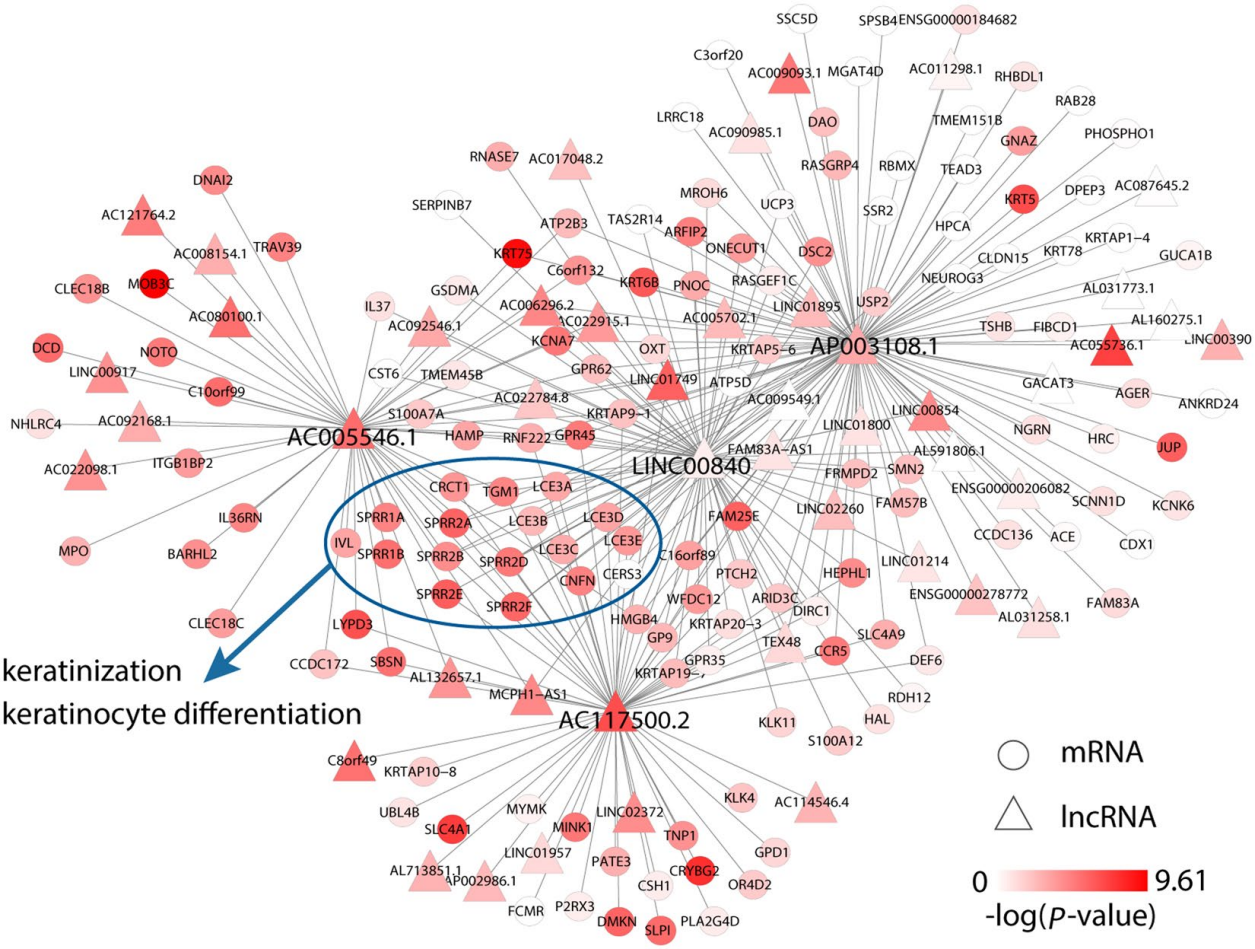
Neighbors of four lncRNAs are enriched on biological processes which included keratinization and keratinocyte differentiation. The circle nodes represent mRNAs and the triangle nodes represent lncRNAs. Genes with a higher −log(*P*-value) were marked dark red. Edges represent the co-expression associations between the four lncRNAs and their neighbors. The edges between neighbors of four lncRNAs are not shown. The mRNAs in blue ellipses indicate genes associated with keratinization and keratinocyte differentiation.

As RRSF tends to select topologically important genes by integrating gene interaction information, we investigated the 10 lncRNAs identified by RRSF at the 10-gene level (Table S4). These 10 lncRNAs are topologically important in the mRNA–lncRNA co-expression network (ranked top 40, Table S4). It has been reported that the functions of lncRNAs could be inferred by their neighbor mRNAs in the mRNA-lncRNA co-expression network^35,36^. After collecting the neighbors for each lncRNA, four were found to be highly connective lncRNAs (*AC117500*.*2*, *AP003108*.*1*, *AC005546*.*1*, and *LINC00840*, Fig. 6) and were enriched on GO biological processes including keratinocyte differentiation (*P* = 2.4 × 10^−16^, 1.3 × 10^−7^, 7.4 × 10^−14^, 8.0 × 10^−15^ for the four lncRNAs, respectively, Benjamini and Hochberg correction) and keratinization (*P* = 3 × 10^−18^, 8.4 × 10^−8^, 1.2 × 10^−13^, 1.1 × 10^−16^ for the four lncRNAs, respectively, Benjamini and Hochberg correction). These two processes are associated with poor survival outcome in several cancers, such as lung squamous cell carcinoma^37^, oropharyngeal squamous cell carcinoma^38^, nasopharyngeal carcinoma^39^, and squamous cell cancer of uterine cervix^40^. Many neighboring mRNAs were found to be common to the four lncRNAs, and were enriched on GO cellular component cornified envelope (*P* = 1.7 × 10^−20^, 6.0 × 10^−8^, 1.4 × 10^−15^, 1.6 × 10^−21^, respectively, Benjamini and Hochberg correction), suggesting that these lncRNAs may work together with their neighboring mRNAs to play important roles in the keratinization process in ESCC development (Fig. 6).

## Discussion

In this study, we proposed a RRSF model to improve predictive performance by integrating gene interaction information into RSF model. RRSF was applied to the survival prediction of patients with GBM and ESCC, respectively. Results showed that, at most levels, RRSF obtained better predictive performance than RSF. Specifically, the RRSF model achieved a favorable performance when the number of genes ranged between eight and 12. Starting from the 10 genes selected by RRSF at the 10-gene level, we identified a two-gene signature for GBM and a three-lncRNA signature for ESCC, which stratified patients into a high-risk group and a low-risk group with significant survival difference for GBM and ESCC, respectively. The prognostic values of these two signatures were verified in independent datasets.

In recent years, gene interaction information has been successfully used to prioritize candidate disease genes^41–43^. The topologically important genes tend to play key roles in disease. In the GBM-TCGA-train dataset, it was found that five of the ten genes identified by RRSF at the 10-gene level possess important functions in mediating GBM cell proliferation and tumor growth. This indicated that RRSF is capable of identifying key disease-associated genes by gradually filtering less important genes out. Although the functions of the five remaining GBM genes have not been reported, the roles of these genes in other cancers are being increasingly studied. For example, *CASP5* has been shown to be a biomarker with diagnostic and therapeutic potential in colorectal cancer^44^, and may act as a suppressor gene in lung cancer with high metastatic potential^45^. *ERP29* over-expression significantly inhibits cell proliferation and suppresses tumorigenesis in breast cancer cells^46^, while its absence is associated with the progression, metastasis, and poor prognosis of gallbladder adenocarcinoma patients^47^. These genes may therefore be potential disease genes for GBM and are worthy of further investigation. In the three-lncRNA signature, *AP003108*.*1* had a degree of 106 in the mRNA-lncRNA co-expression network. It co-expressed with a lot of differential mRNAs involved in many biological functions (Fig. 6), thus suggesting that it has an important role in ESCC development. *MAMDC2-AS1*, and *AC146944*.*4* had node degrees of 3, and 5, respectively. Li *et al*. previously identified a three-lncRNA signature (*ENST00000435885*.*1*, *XLOC_013014* and *ENST00000547963*.*1*), which obtained similar predictive performance on the three ESCC datasets^15^. However, this signature provided limited biological interpretation. None of the three lncRNAs co-expressed with any differential mRNAs. This indicates that the three-lncRNA signature identified by RRSF had a higher biological relevance with ESCC.

In the RRSF model, there are several adjustable parameters. The first is the Cox P-value cutoff used to select the initial gene sets. Using a larger cutoff will incorporate a large number of genes with limited discriminative power into the RRSF model. This may reduce algorithm efficiency and increase noise in the RRSF model. On the contrary, using a smaller cutoff value may result in the loss of some important discriminative information. In this study, we used a moderate cutoff of 0.05 as it resulted in a better overall predictive performance on both the GBM (Fig. S14) and ESCC datasets (Fig. S15) than a more relaxed cutoff of 0.1 and a stricter cutoff of 0.01. The second adjustable parameter is the initial weights used in the DRW algorithm. Proving effective in previous studies^13,48^, we set the initial weights based on *P*-values from univariate Cox regression analysis. Two alternative strategies were also tested. One is based on VIMP from a standard RSF analysis, while the other uses uniform initial weights. Results showed that the initial weights based on Cox *P*-value resulted in better predictive performance than the two alternative options on both GBM (Fig. S16) and ESCC (Fig. S17) datasets. The third parameter that can be adjusted is the source of the interaction information. The accuracy of the interaction information contributes towards the accurate evaluation of topological importance^48^, ultimately influencing the predictive performance of RRSF. Compared to global pathway network analysis, a less stringent co-expression network may impact the predictive performance^48^. This phenomenon was observed in the GBM datasets, where the predictive performance of the RRSF model using a co-expression network as gene interaction information was reduced (Fig. S18). The fourth adjustable parameter is the method used to evaluate the importance of genes during the gene filtering process (Fig. 1C). When we used minimal depth^49^ instead of VIMP to evaluate the importance of genes, comparable predictive performance on both GBM (Fig. S19) and ESCC (Fig. S20) datasets was obtained. Thus, minimal depth is an alternative method for filtering features in RRSF.

As with other methods, such as DRWPClass^9^, RPCR^13^, and DRWPSurv^48^, which incorporate gene interaction information for binary classification or survival prediction, RRSF also possesses the characteristic of good generalization. It yielded C-indices of 0.58–0.63 on two independent GBM datasets; these results being better than those obtained for the GBM-TCGA-test dataset (Fig. S3). RRSF models based on 100 times random partition of GBM-TCGA obtained similar predictive performance between test set and two independent datasets (Fig. S21), indicating that the generalization performance was not due to the specific partition of GBM-TCGA dataset. The two-gene biomarker also showed good generalization performance (Fig. S22). It gave a better stratification of GBM patients in the GSE4412 (log-rank P-value: 0.00053 vs 0.0137) and GSE4271 (0.00037 vs 0.0461) datasets than RPCR, respectively. Compared to RPCR^13^, DRWPSurv^48^, and other pathway-based survival prediction methods^50,51^, which focus on identifying important pathways and need a large number of differential genes to achieve accurate prediction, RRSF has the unique advantage in that it can be used for feature selection and biomarker identification. This makes RRSF convenient for clinical application. We also compared the predictive performance of our two-gene and three-lncRNA signatures with gene signatures identified by minimal depth, which is a prevalent feature selection method for high-dimensional survival data^49^. The gene signatures identified by minimal depth failed to give stratifications with significant differences in survival outcome on GBM patients in the GSE4412 dataset (Fig. S23) and ESCC patients in the ESCC-test and ESCC-valid (Fig. S22) datasets.

In this study, we showed that, by integrating gene interaction information, RRSF could yield better predictive performance based not only on mRNA expression data (GBM as an example), but on lncRNA expression data as well (ESCC as an example). In fact, the predictive performance can be further improved if both lncRNA and mRNA data are used (Fig. S25). RRSF can be easily transferred to the survival prediction of patients with other cancers if mRNA and/or lncRNA expression data and precise gene interaction are available.

In all, the integration of gene interaction information assists RRSF in selecting biologically meaningful gene markers and predicts survival outcome with better accuracy. However, gene interaction information is far from complete, especially for lncRNAs. With more gene interaction data available, we believe that RRSF will benefit from the precise interaction information and yield better predictive performance. The RRSF method is implemented as an R package “RRSF”, which is freely available at https://github.com/weiliu123/RRSF-package.

## Electronic supplementary material

Supplementary figures

Supplementary tables
