# Supplementary material for: Integration of gene interaction information into a reweighted random survival forest approach for accurate survival prediction and survival biomarker discovery
Source: Sci Rep. 2018 Sep 4;8:13202. doi: 10.1038/s41598-018-31497-0 (PMC6123437; doi:10.1038/s41598-018-31497-0)
Supplement: Supplementary file 1 — Supplementary figures [file 41598_2018_31497_MOESM1_ESM.docx]

**Integration of gene interaction information into a reweighted random survival forest approach for accurate survival prediction and survival biomarker discovery**

Wei Wang^a^, Wei Liu^a,b^

^a^Department of Mathematics, Heilongjiang Institute of Technology, Harbin, 150050, China.

^b^The Key Laboratory of Molecular Biology for High Cancer Incidence Coastal Chaoshan Area, Shantou University Medical College, Shantou, 515041, China.

Correspondence and requests for materials should be addressed to W.L. (freelw@qq.com)

Supplementary Figures


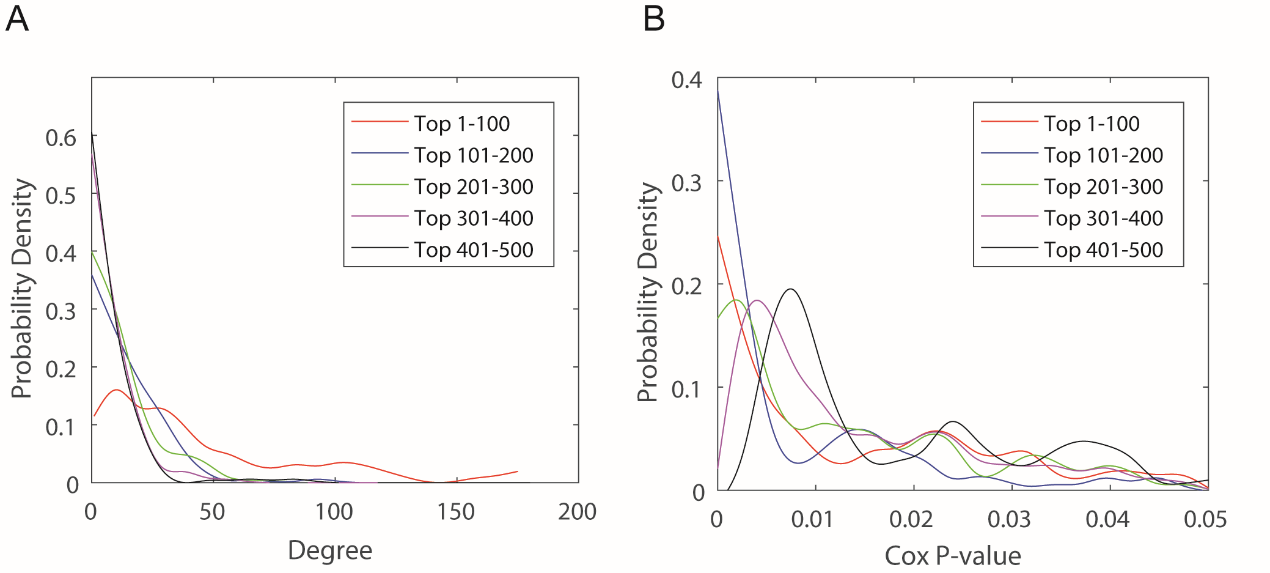


**Figure S1. Topological weights reflect topological importance of genes.** (**A**) Degree distribution. Genes with a large degree tend to have large topological weights. (**B**) Cox *P*-value distribution. Genes with a small Cox *P*-value tend to have large topological weights.


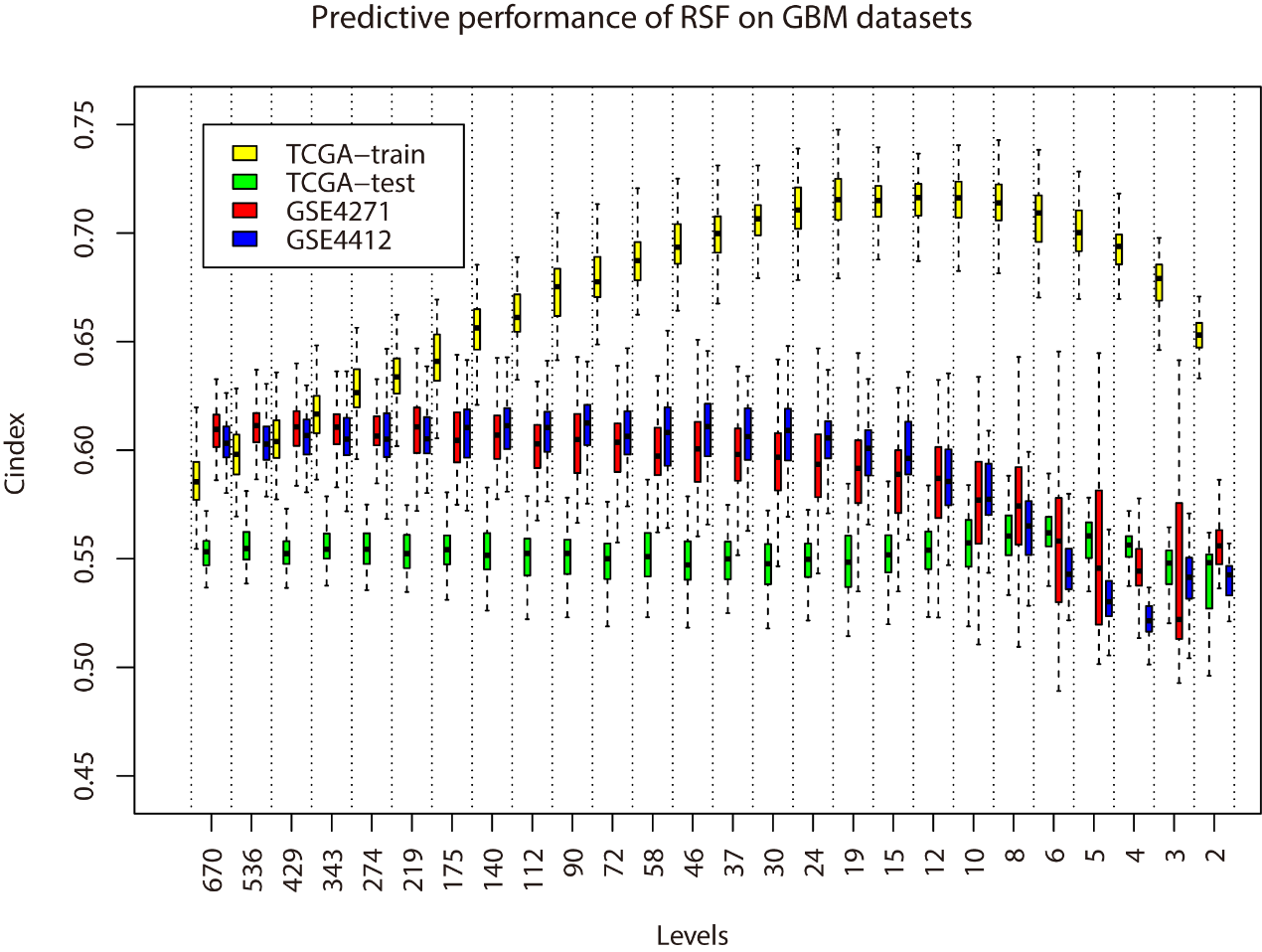


**Figure S2. Predictive performance of RSF on GBM datasets.**


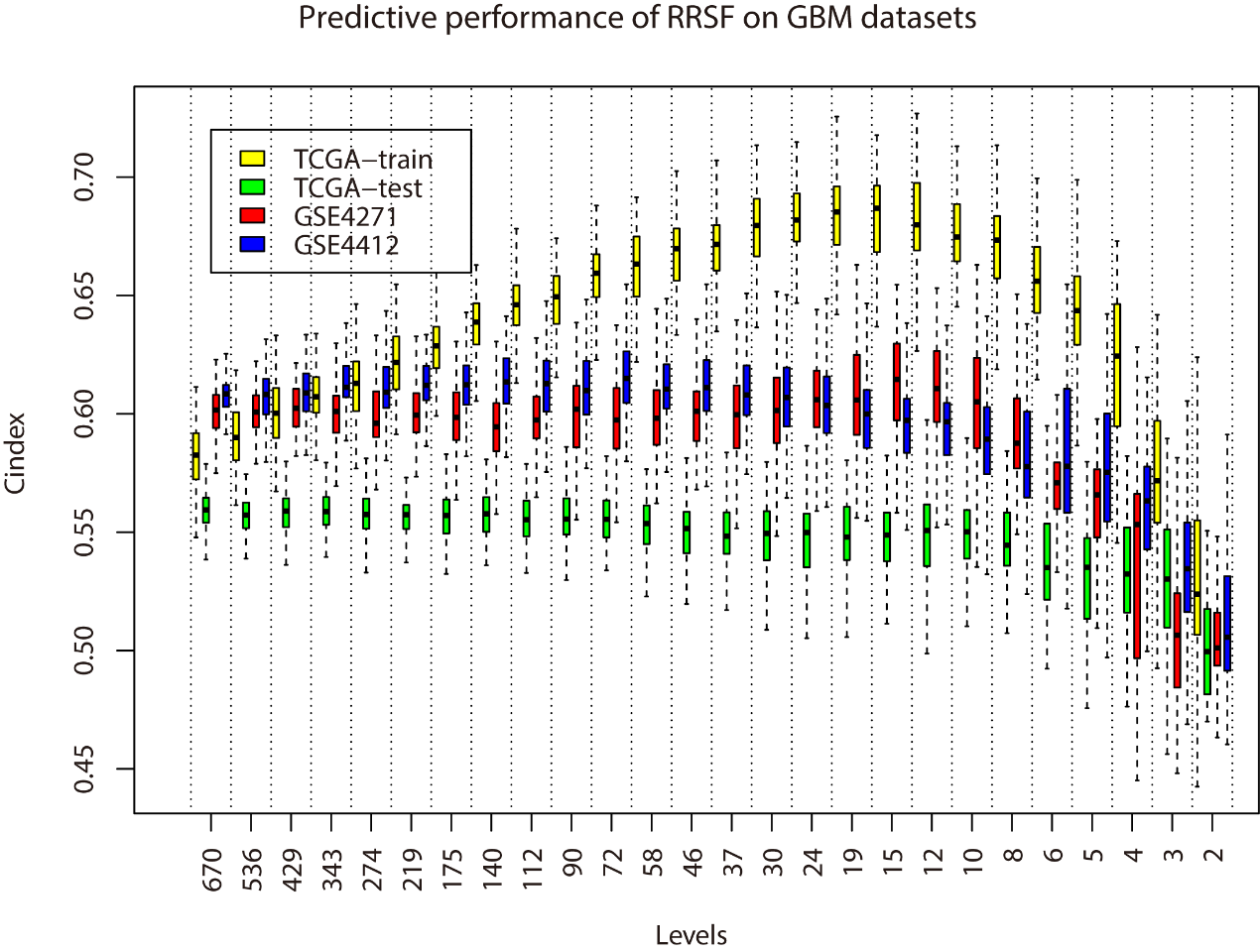


**Figure S3. Predictive performance of RRSF on GBM datasets.**


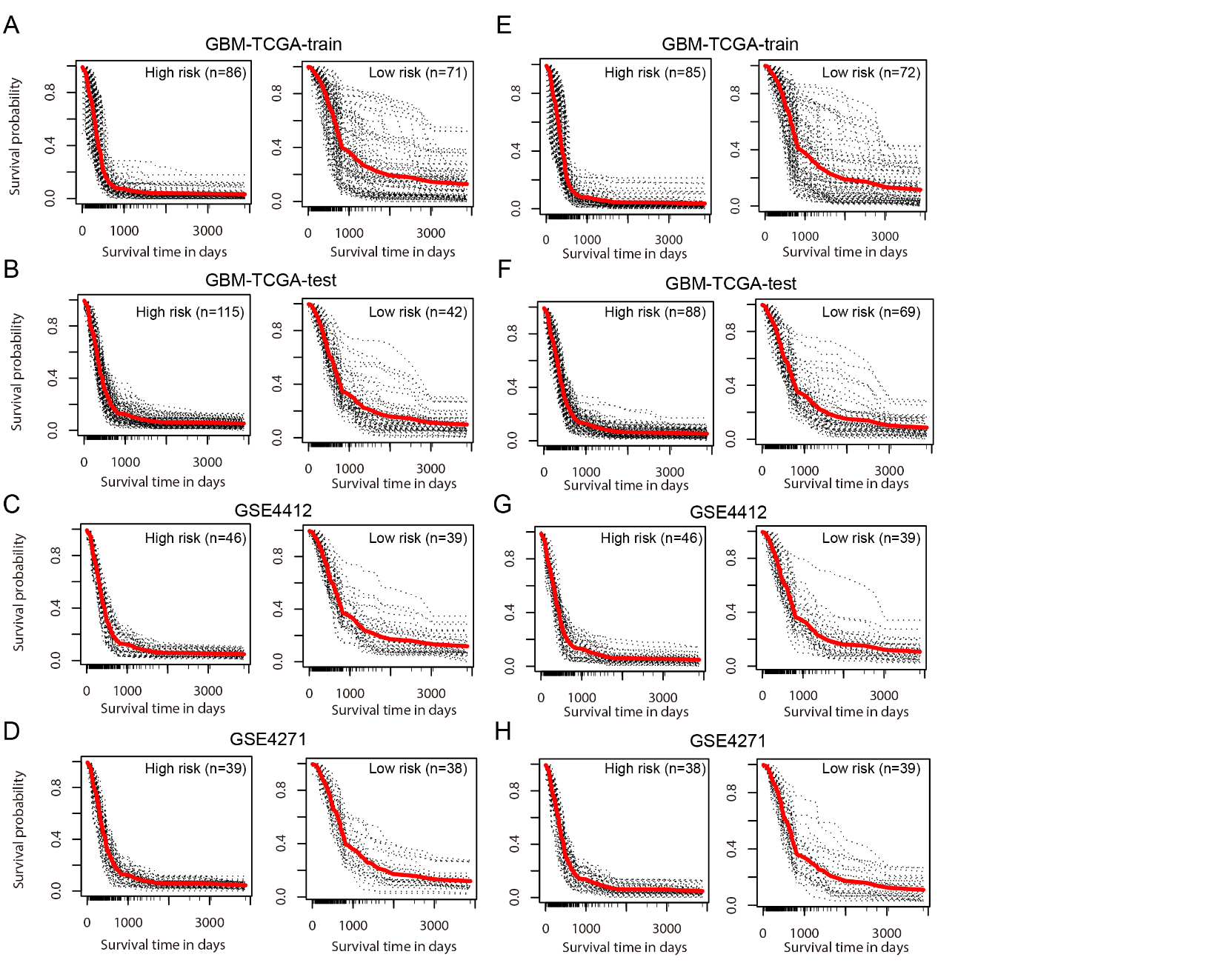


**Figure S4. Forest estimated survival function comparison of RRSF and RSF at 10-gene level on GBM datasets.** GBM patients were stratified into high- and low-risk groups using the mean of predicted values of patients in GBM-TCGA-train as the cutoff. In each group (high risk and low risk), survival curves of forest estimated survival function for each individual were shown. Thick red line was overall ensemble survival. (**A**-**D**) RRSF, (**E**-**H**) RSF.


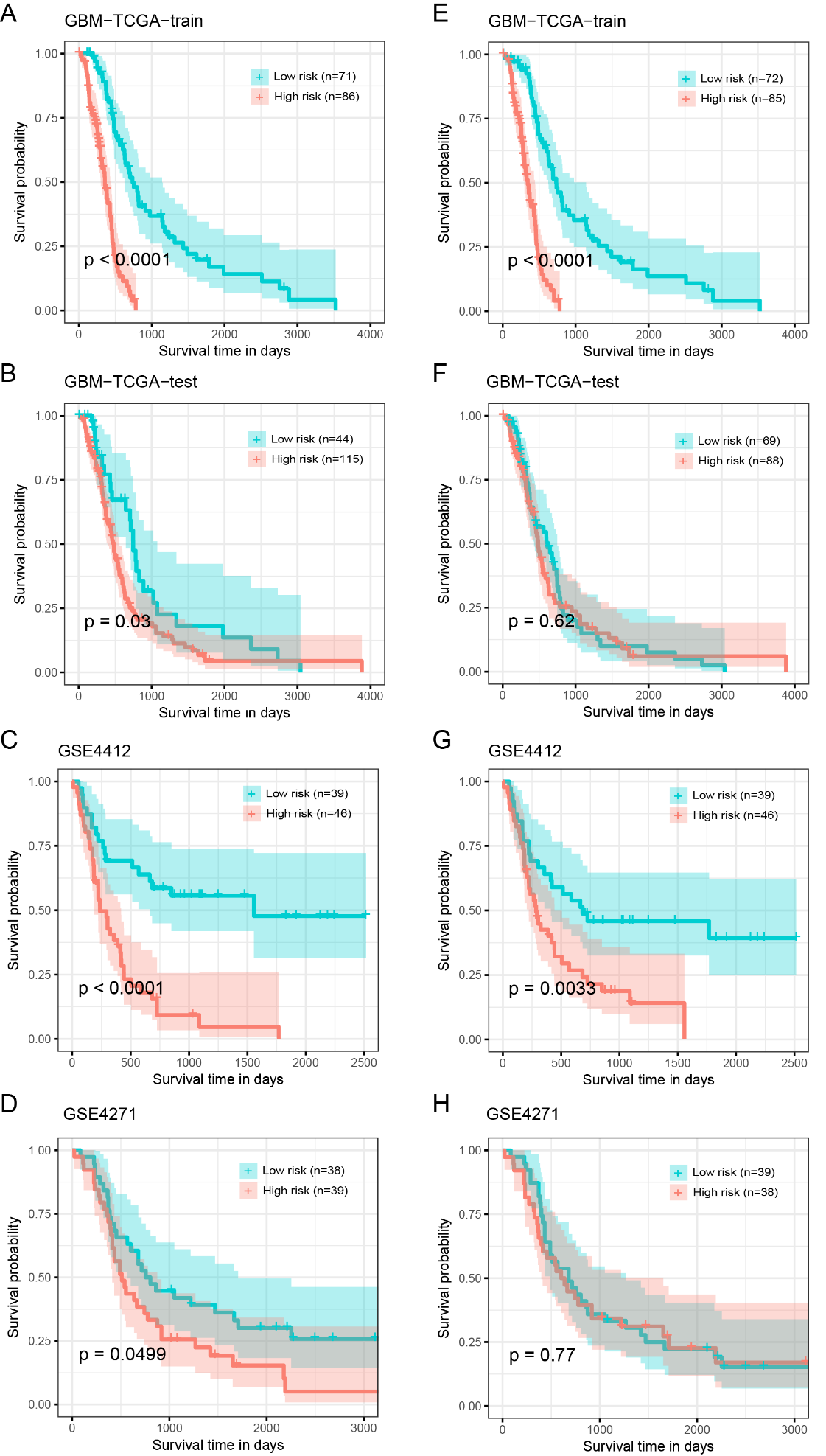


**Figure S5. Kaplan-Meier survival curves comparison of RRSF and RSF at 10-gene level on GBM datasets.** The predicted values of patients were calculated by RRSF/RSF model at 10-gene level. GBM patients were stratified into high- and low-risk groups using the mean of predicted values of patients in GBM-TCGA-train as the cutoff. P values were calculated by log-rank test. (**A**-**D**) RRSF, (**E**-**H**) RSF.


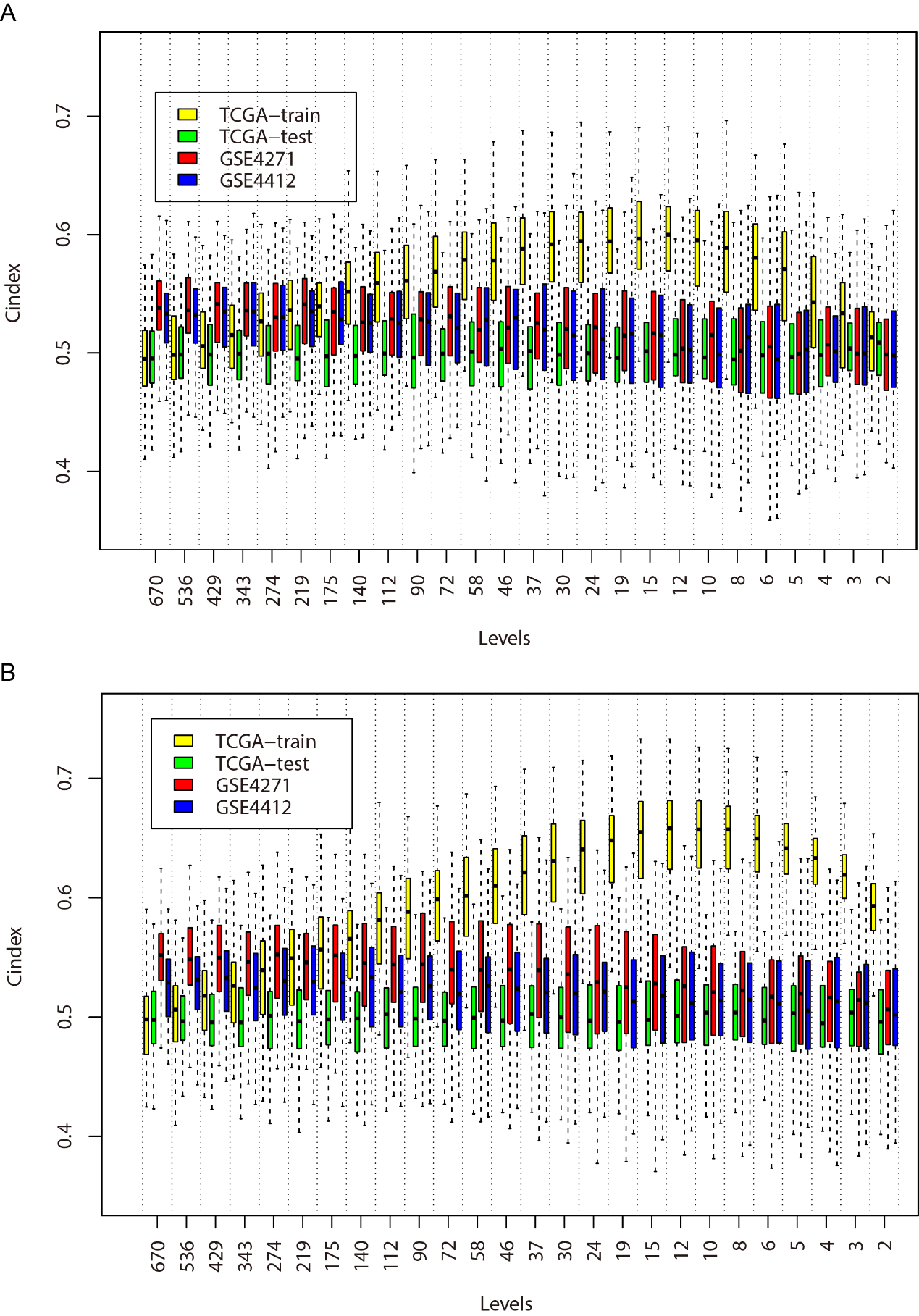


**Figure S6. Evaluation of false positive findings of RRSF and RSF on GBM datasets.** RRSF and RSF models were trained on TCGA-train with permuted survival outcome. The predictive performance on TCGA-test, GSE4271, and GSE4412 was not better than random. (**A**) RRSF, (**B**) RSF.


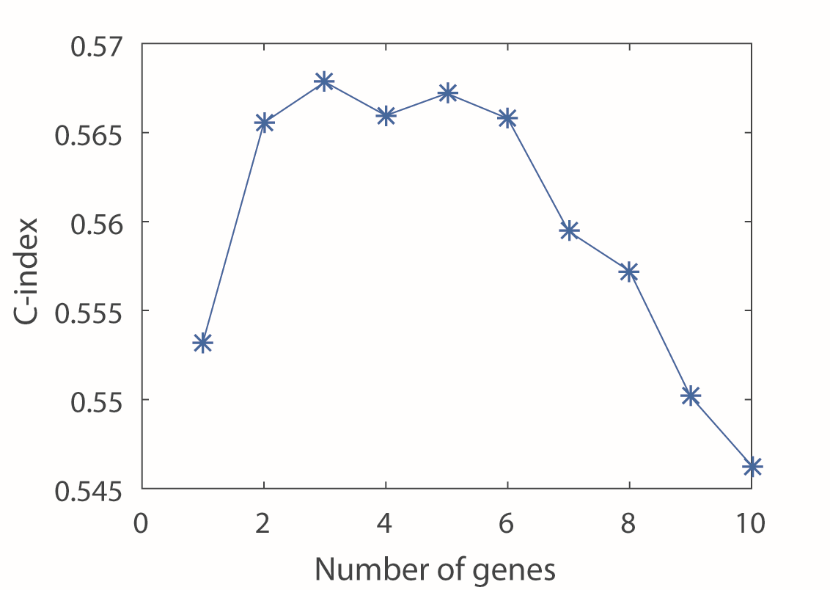


**Figure S7. The procedure for identifying the two-gene signature.** The C-indexes of all 1023 signatures on GBM-TCGA-test were calculated and the 10 average C-indexes for k=1, 2, …, 10 are shown in the plot. The signature containing two genes was selected as the final signature.


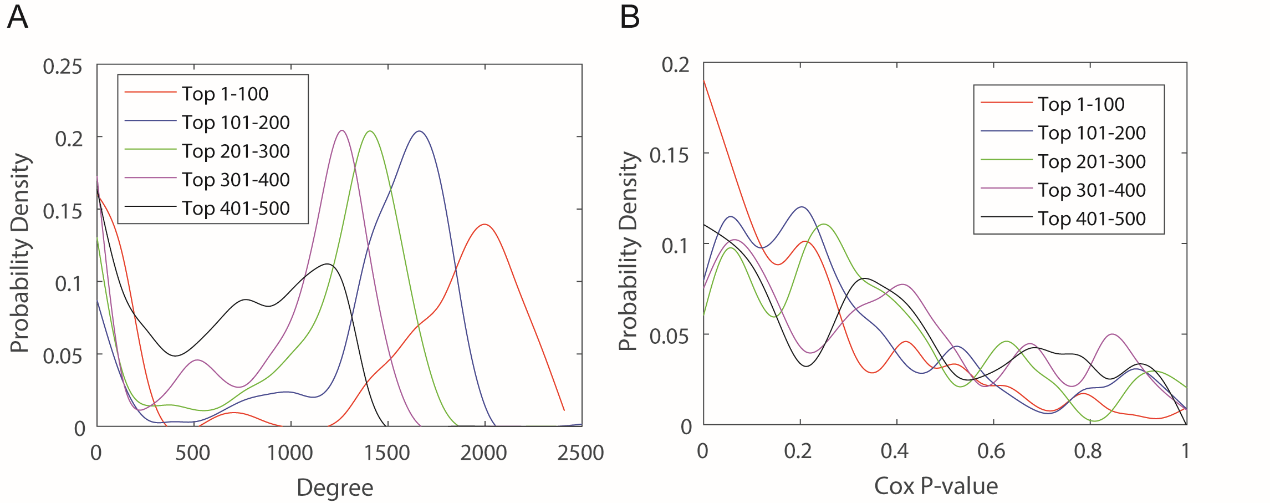


**Figure S8. Topological weights reflect topological importance of lncRNAs.** (**A**) Degree distribution. LncRNAs with a large degree tend to have large topological weights. (**B**) Cox *P*-value distribution. LncRNAs with a small Cox *P*-value tend to have large topological weights.


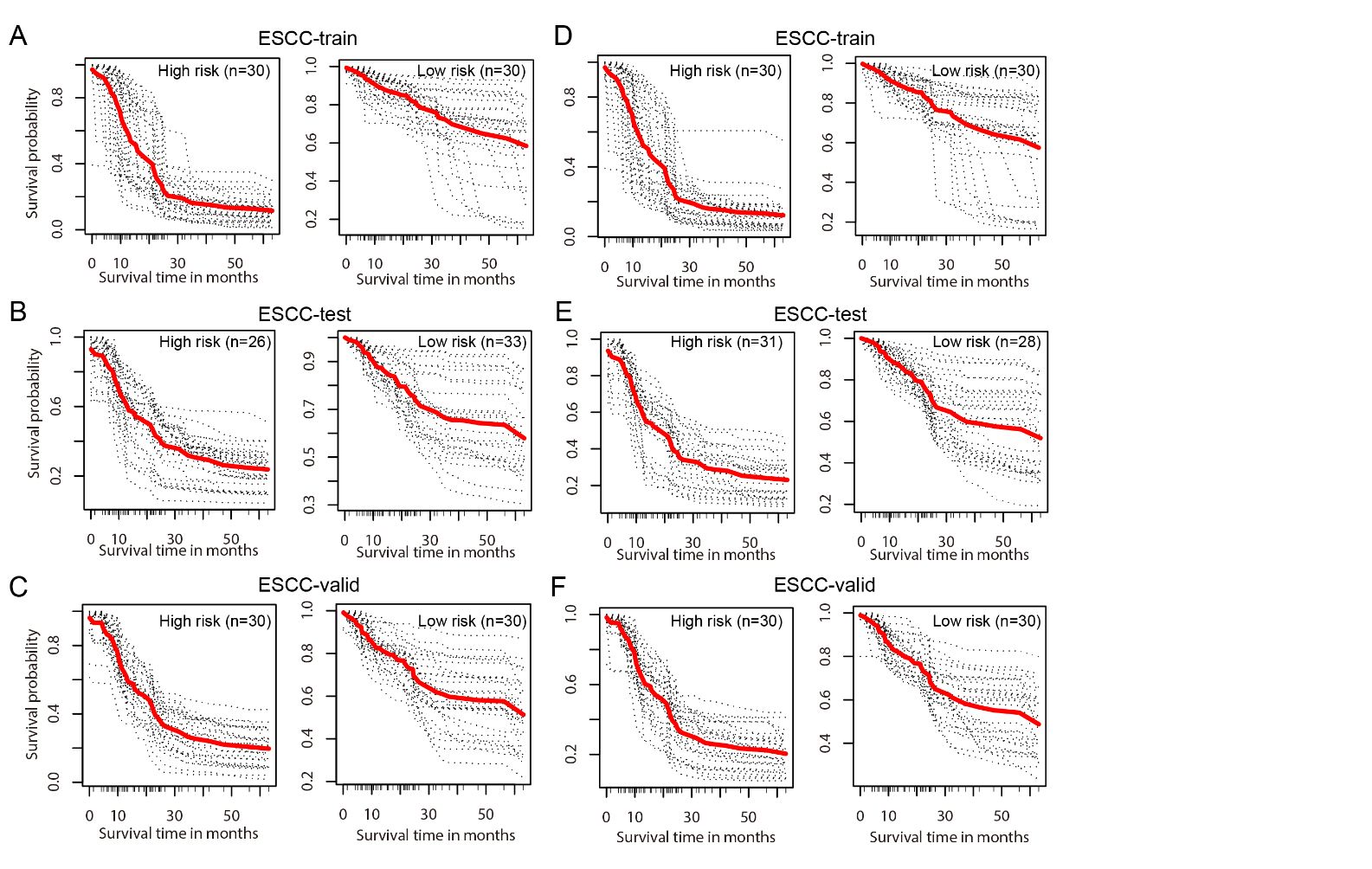


**Figure S9. Forest estimated survival function comparison of RRSF and RSF at 10-gene level on ESCC datasets.** ESCC patients were stratified into high- and low-risk groups using the mean of predicted values of patients in ESCC-train as the cutoff. In each group (high risk and low risk), survival curves of forest estimated survival function for each individual were shown. Thick red line was overall ensemble survival. (**A**-**C**) RRSF, (**D**-**F**) RSF.


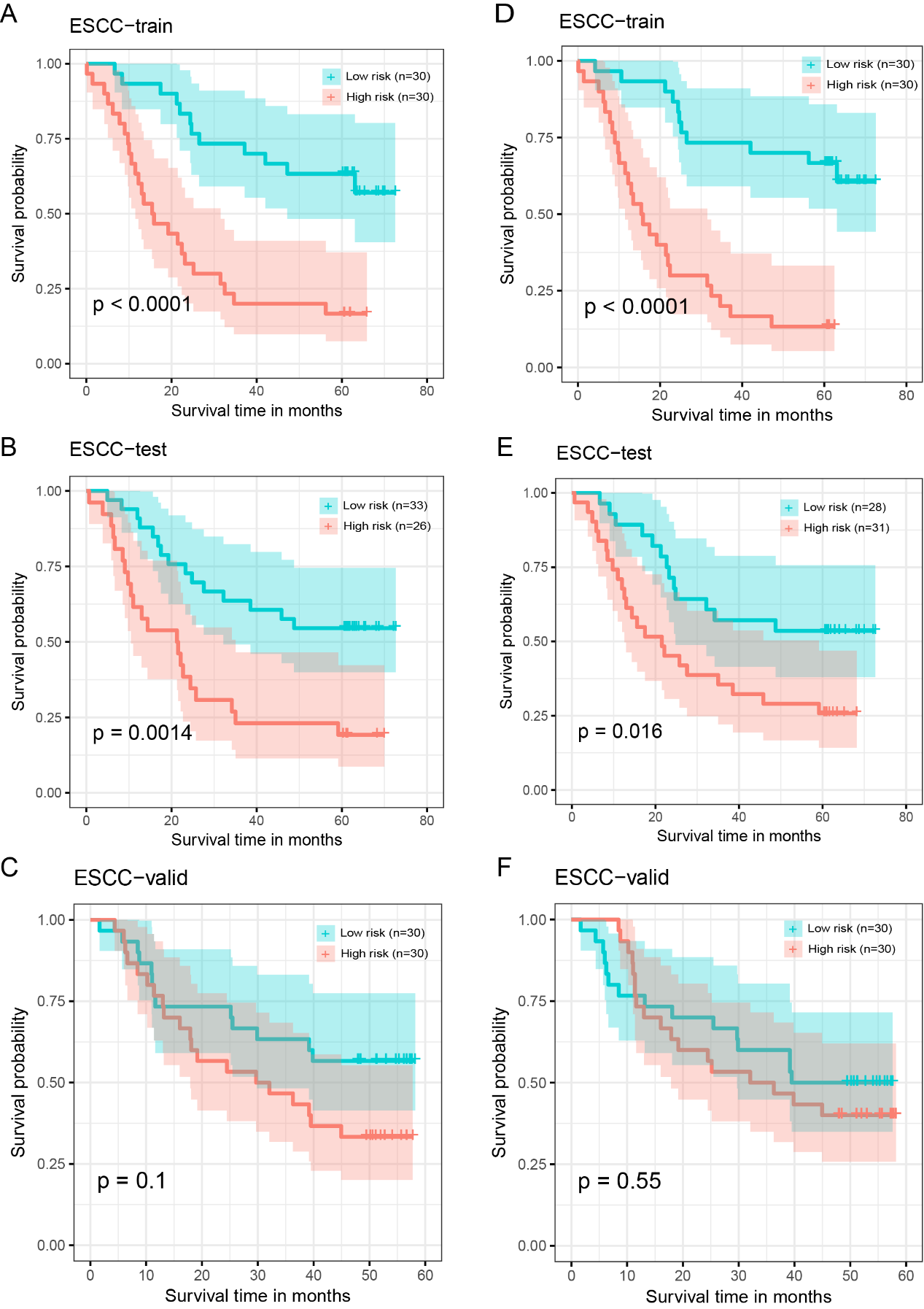


**Figure S10. Kaplan-Meier survival curves comparison of RRSF and RSF at 10-gene level on ESCC datasets.** The predicted values of patients were predicted by RRSF/RSF model at 10-gene level. ESCC patients were stratified into high- and low-risk groups using the mean of the predicted values of patients in ESCC-train as the cutoff. P values were calculated by log-rank test. (**A**-**C**) RRSF, (**D**-**F**) RSF.


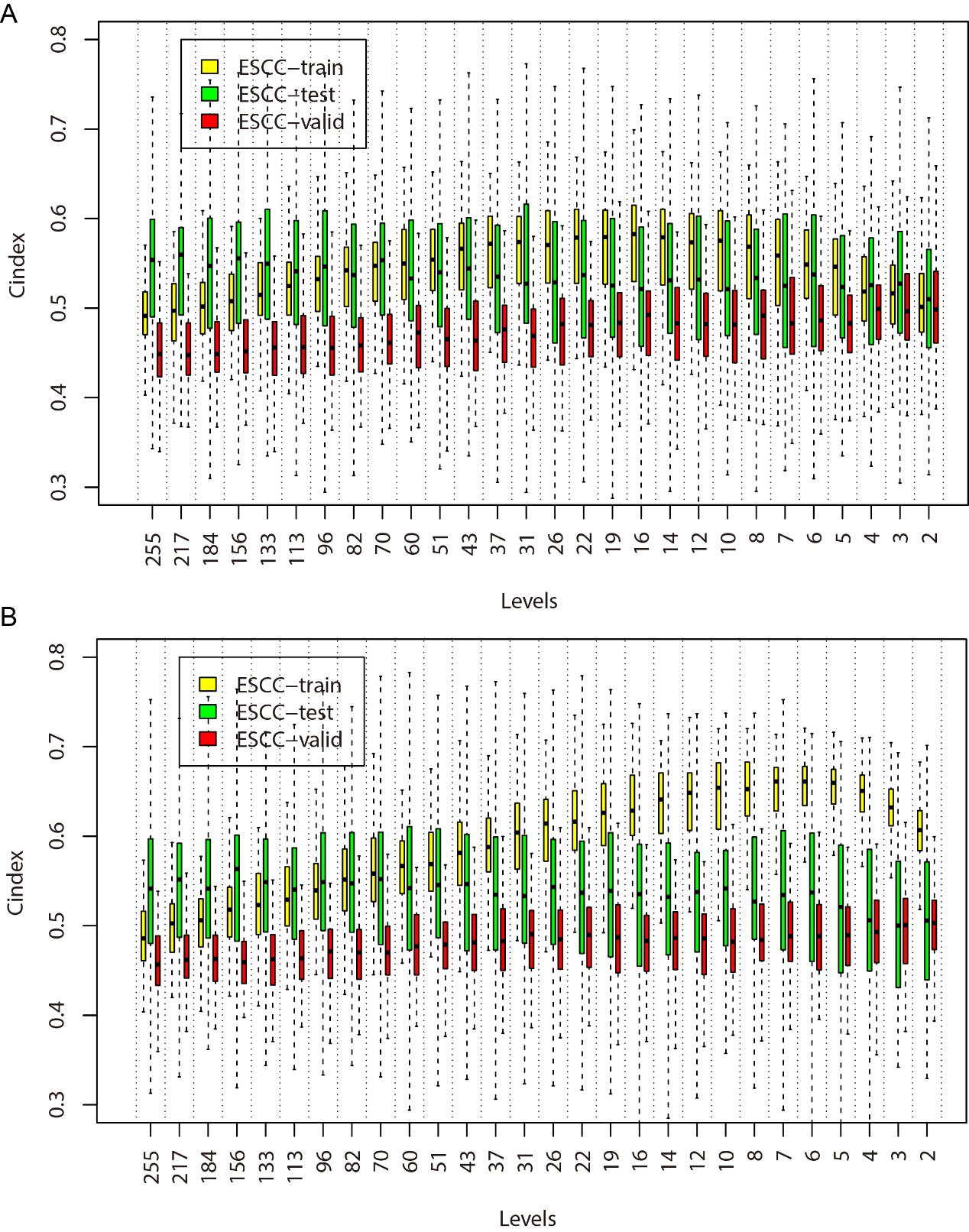


**Figure S11. Evaluation of false positive findings of RRSF and RSF on ESCC datasets.** RRSF and RSF models were trained on ESCC-train with permuted survival outcome. The predictive performance on ESCC-test and ESCC-valid was not better than random. (**A**) RRSF, (**B**) RSF.


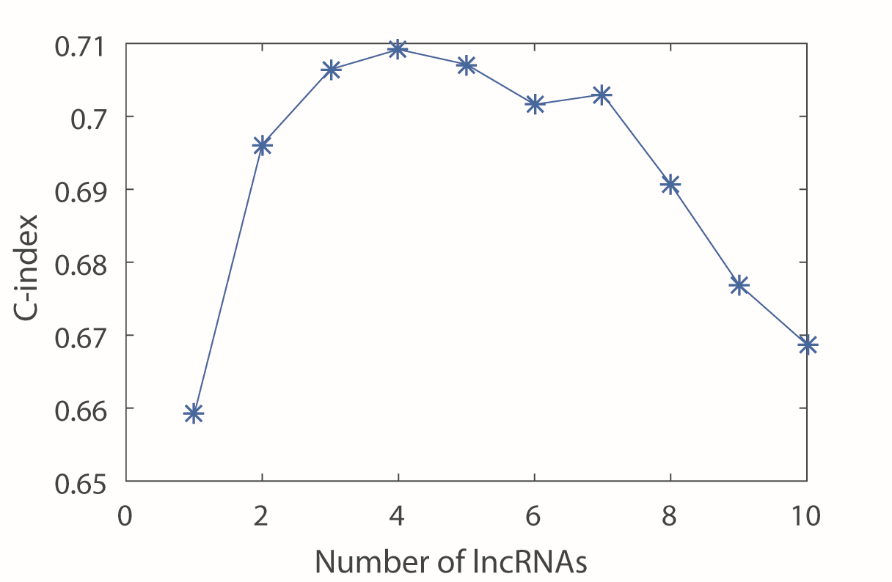


**Figure S12. The procedure for identifying the three-lncRNA signature.** The C-indexes of all 1023 signatures on ESCC-test were calculated and the 10 average C-indexes for k=1, 2, …, 10 are shown in the plot. The signature containing three lncRNAs was selected as the final signature.


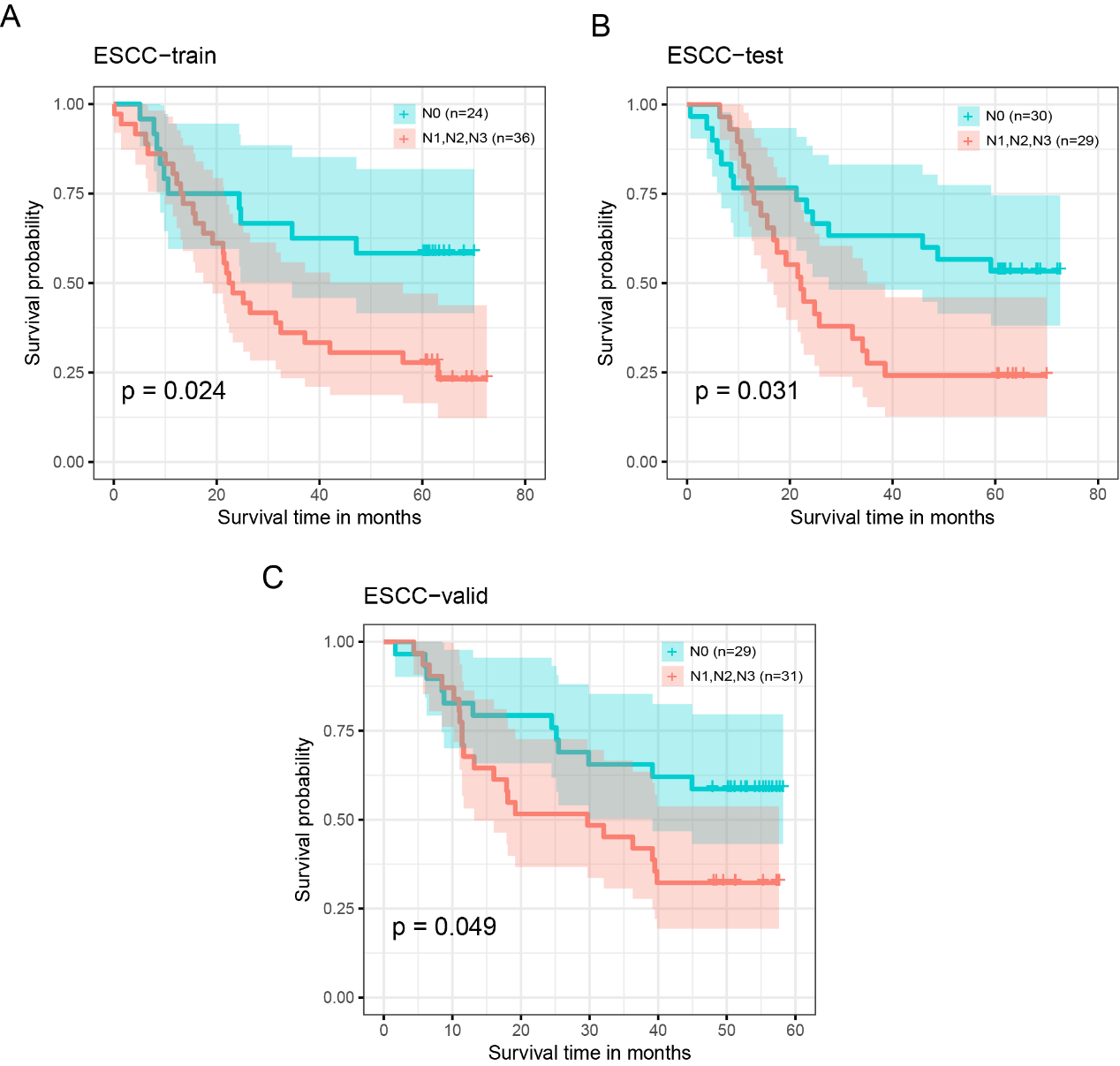


**Figure S13. Stratification of ESCC patients based on N-stage.** ESCC patients were stratified into a high-risk group (N1, N2, and N3) and a low-risk group (N0). (**A**) ESCC-train, (**B**) ESCC-test, and (**C**) ESCC-valid. P values were calculated by log-rank test.


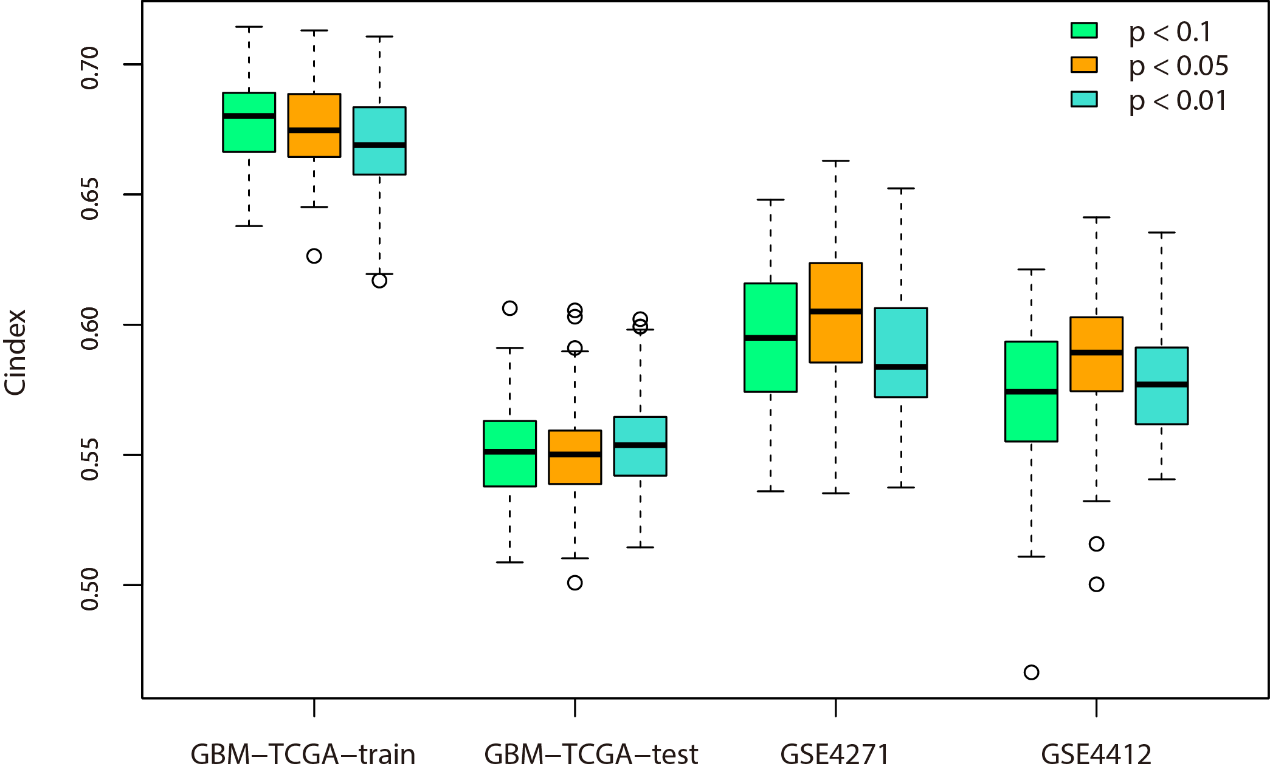


**Figure S14. Predictive performance of RRSF on GBM datasets with genes selected according to different cutoff.** Shown are the C-indexes at 10-gene level. A total of 983, 670, and 246 genes were selected as the initial feature set for Cox P-value < 0.1, 0.05, and 0.01, respectively. No cutoff was consistently better than others. The cutoff 0.05 had slightly better overall performance.


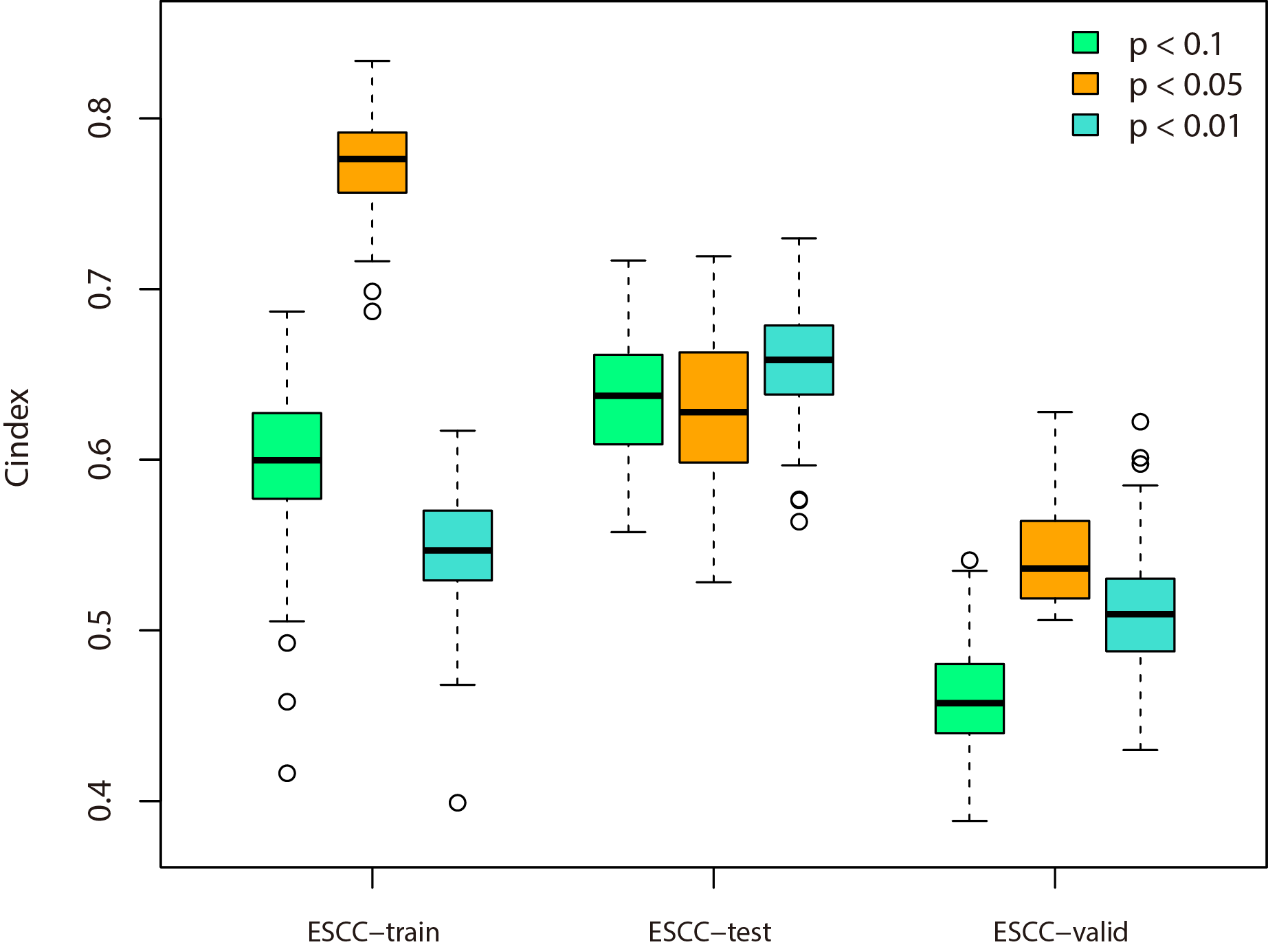


**Figure S15. Predictive performance of RRSF on ESCC datasets with genes selected according to different cutoff.** Shown are the C-indexes at 10-gene level. A total of 499, 255, and 62 genes were selected as the initial feature set for Cox P-value < 0.1, 0.05, and 0.01, respectively. No cutoff was consistently better than others. The cutoff 0.05 had slightly better overall performance.


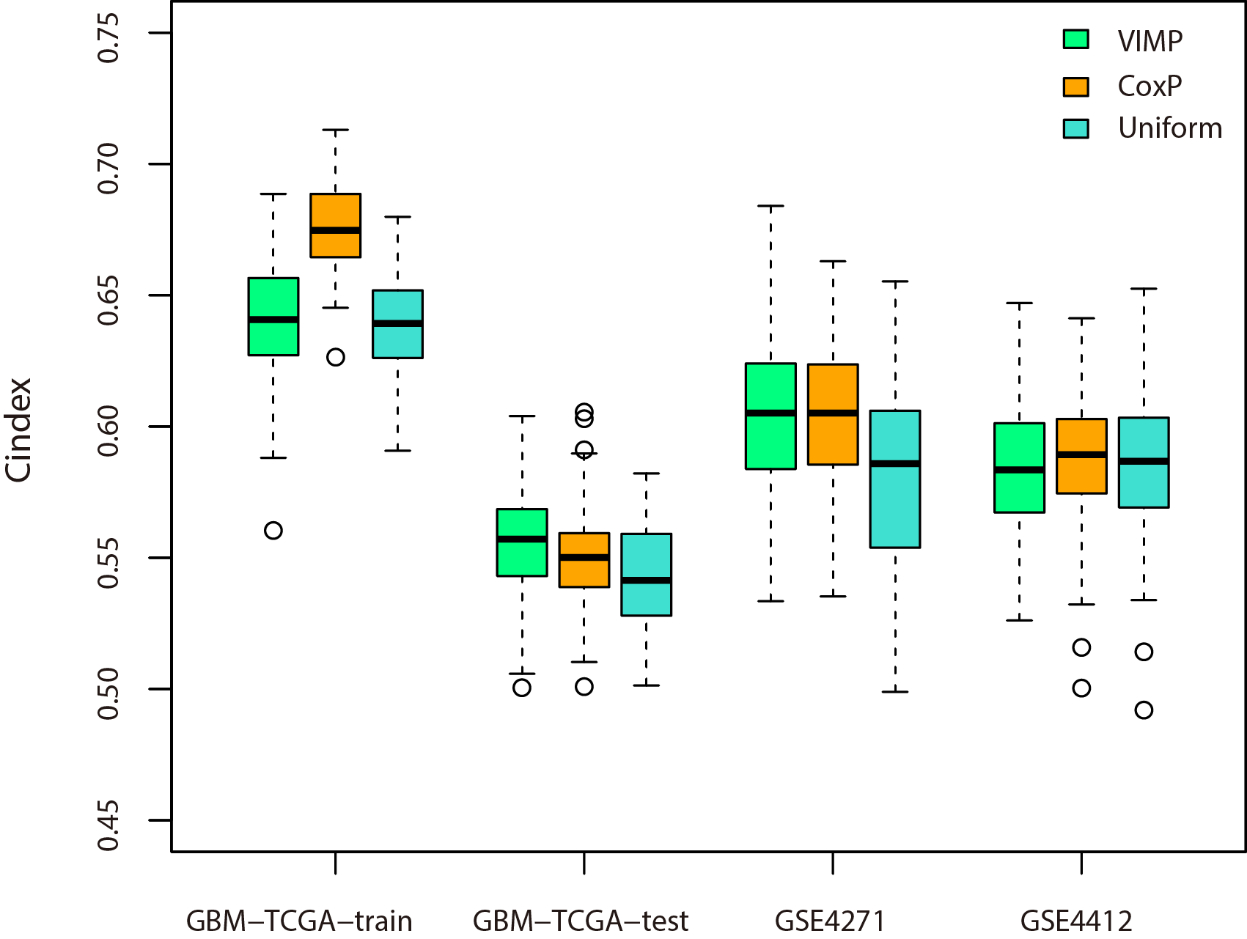


**Figure S16. Predictive performance of RRSF on GBM datasets with different initial weights.** Shown are the C-indexes at 10-gene level. VIMP, the initial weights in DRW algorithm were set based on the variable importance calculated using a standard RSF algorithm. CoxP, the initial weights were set based on Cox *P*-values. Uniform, the initial weights were uniform.


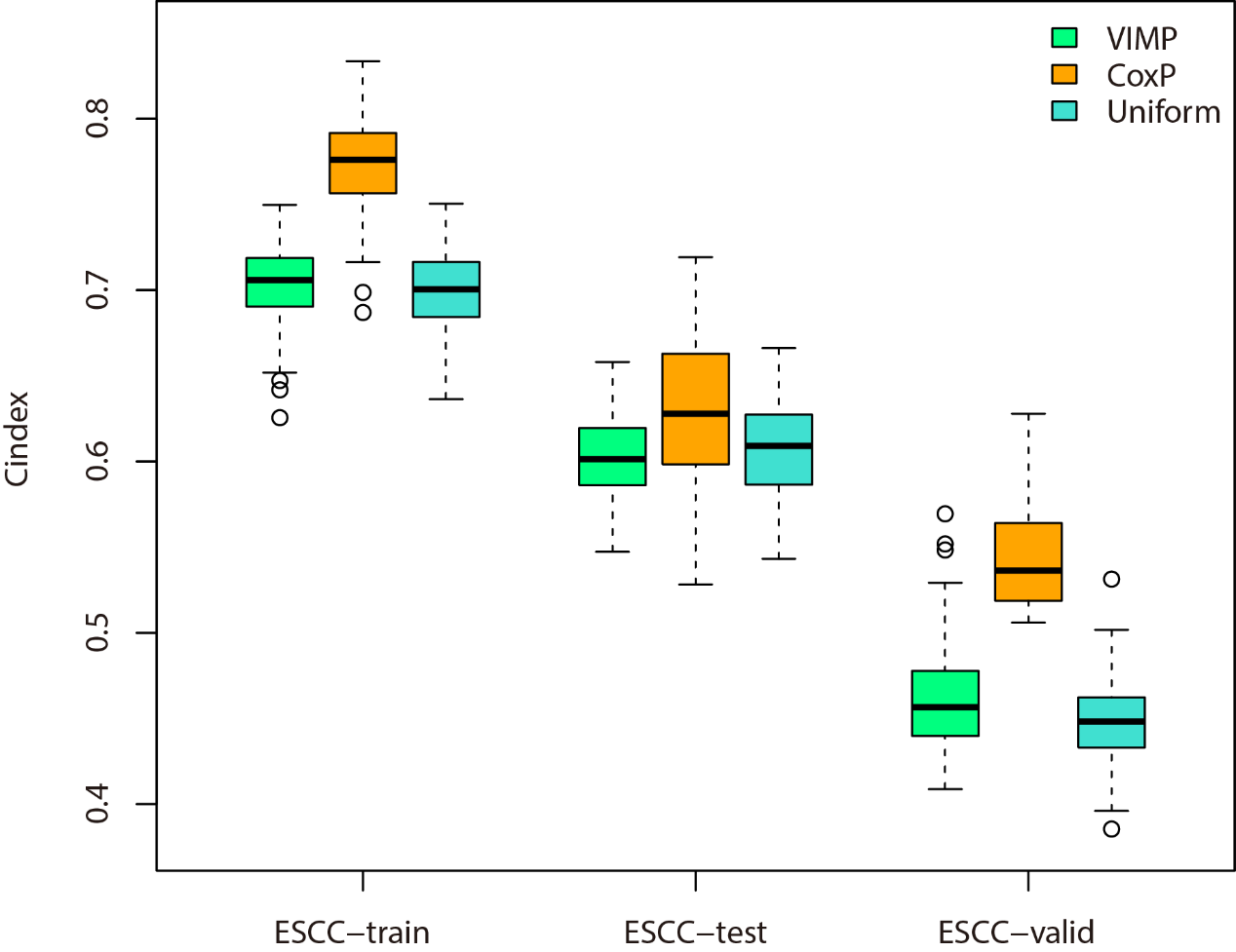


**Figure S17. Predictive performance of RRSF on ESCC datasets with different initial weights.** Shown are the C-indexes at 10-gene level. VIMP, the initial weights in DRW algorithm were set based on the variable importance calculated using a standard RSF algorithm. CoxP, the initial weights were set based on Cox *P*-values. Uniform, the initial weights were uniform.


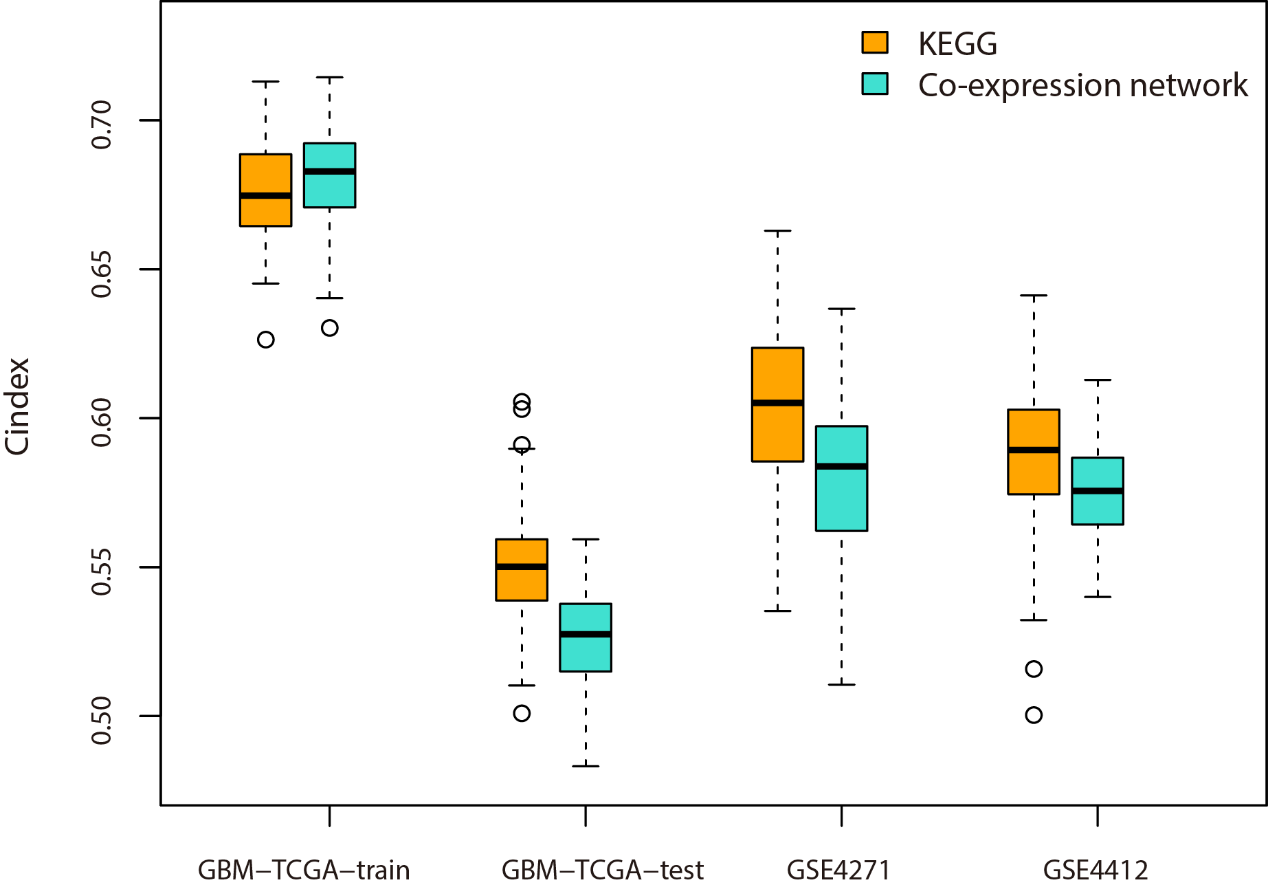


**Figure S18. Predictive performance of RRSF on GBM datasets with different gene interaction information.** Shown are the C-indexes at 10-gene level. KEGG, RRSF with KEGG pathway network as gene interaction information. Co-expression network, RRSF with gene co-expression network as gene interaction information.


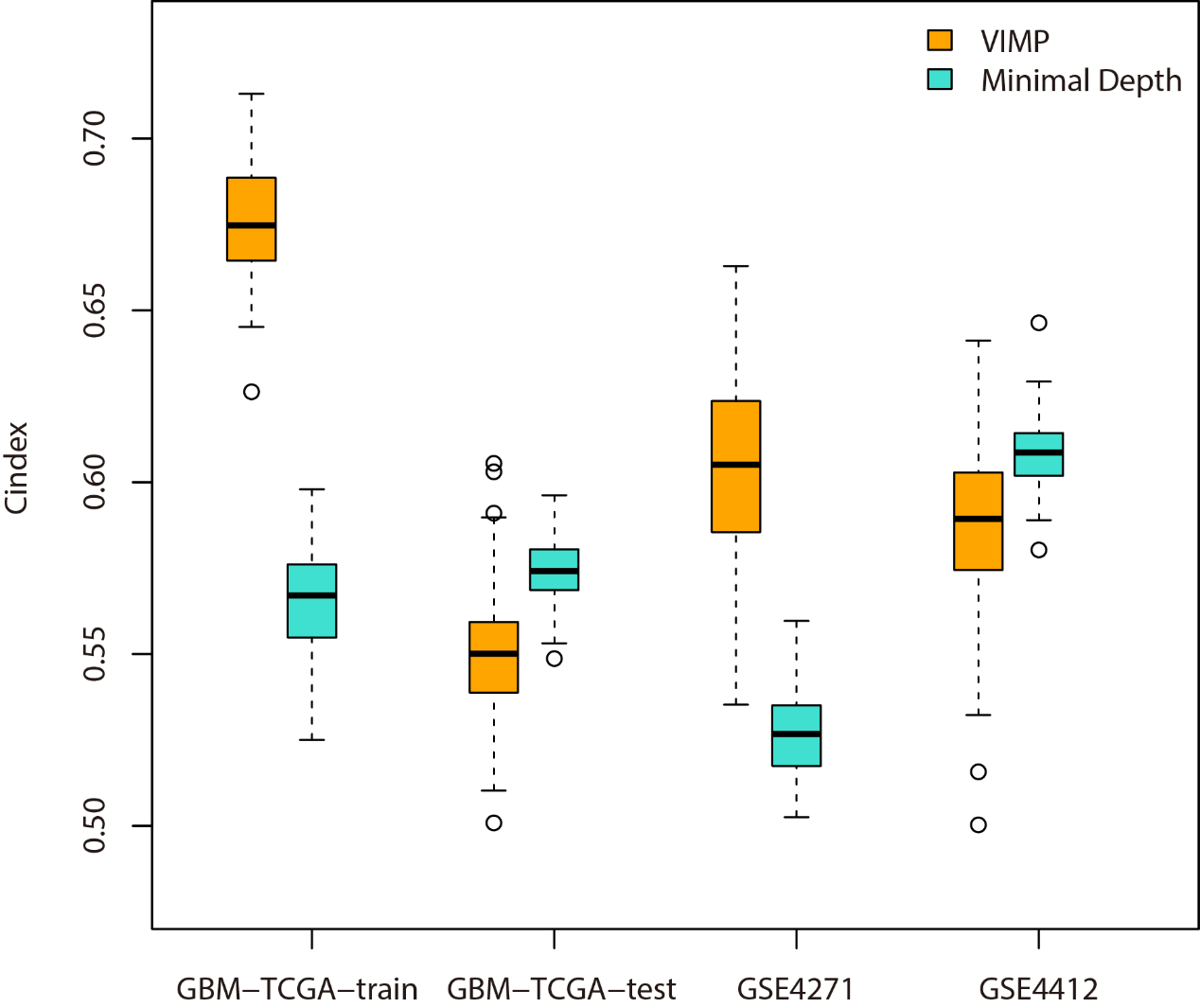


**Figure S19. Predictive performance of RRSF on GBM datasets with different feature selection methods.** Shown are the C-indexes at 10-gene level. VIMP, discard 10% less important genes based on VIMP of genes. Minimal Depth, discard 10% less important genes based on minimal depths of genes.


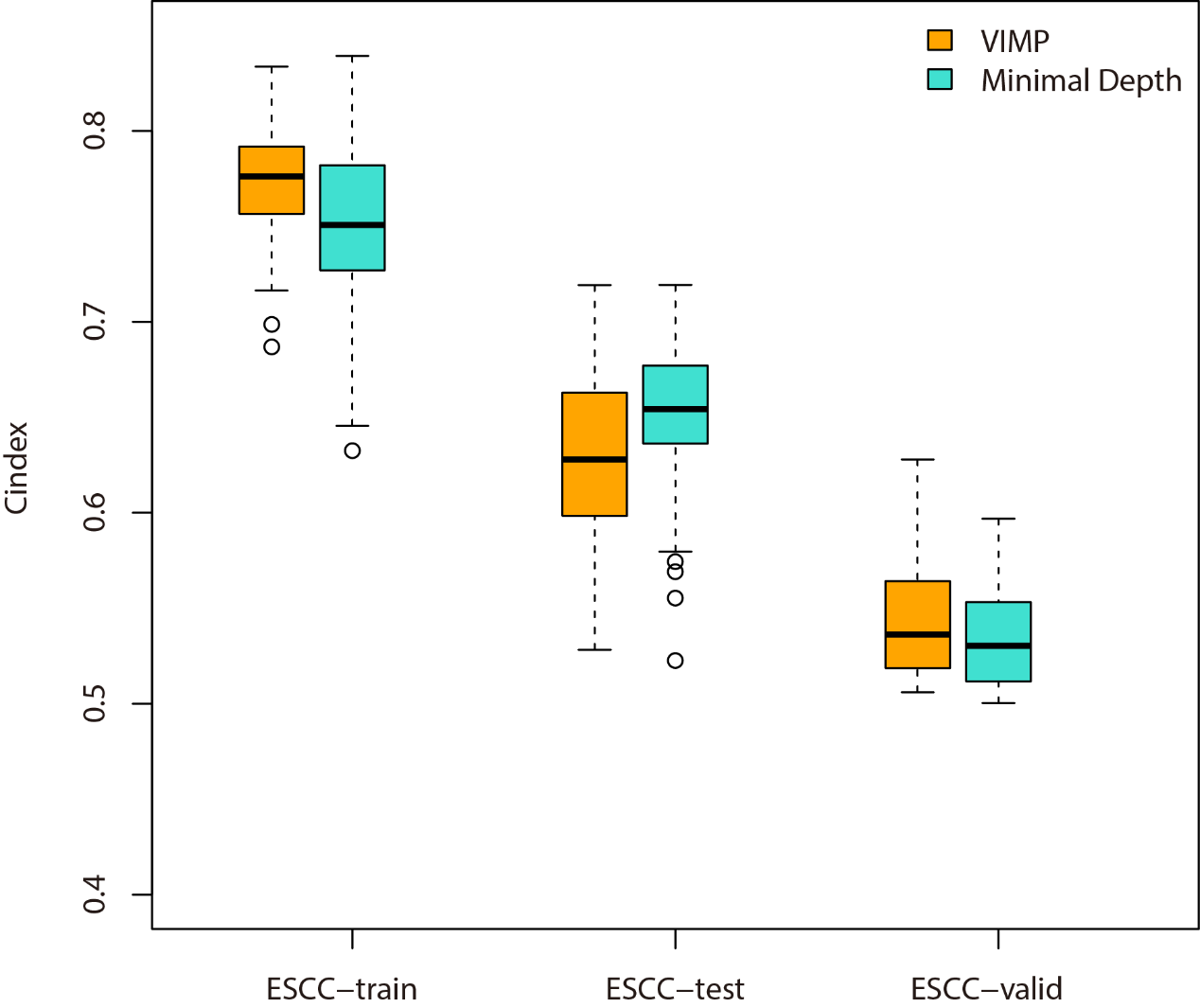


**Figure S20. Predictive performance of RRSF on ESCC datasets with different feature selection methods.** Shown are the C-indexes at 10-gene level. VIMP, discard 10% less important genes based on VIMP of genes. Minimal Depth, discard 10% less important genes based on minimal depths of genes.


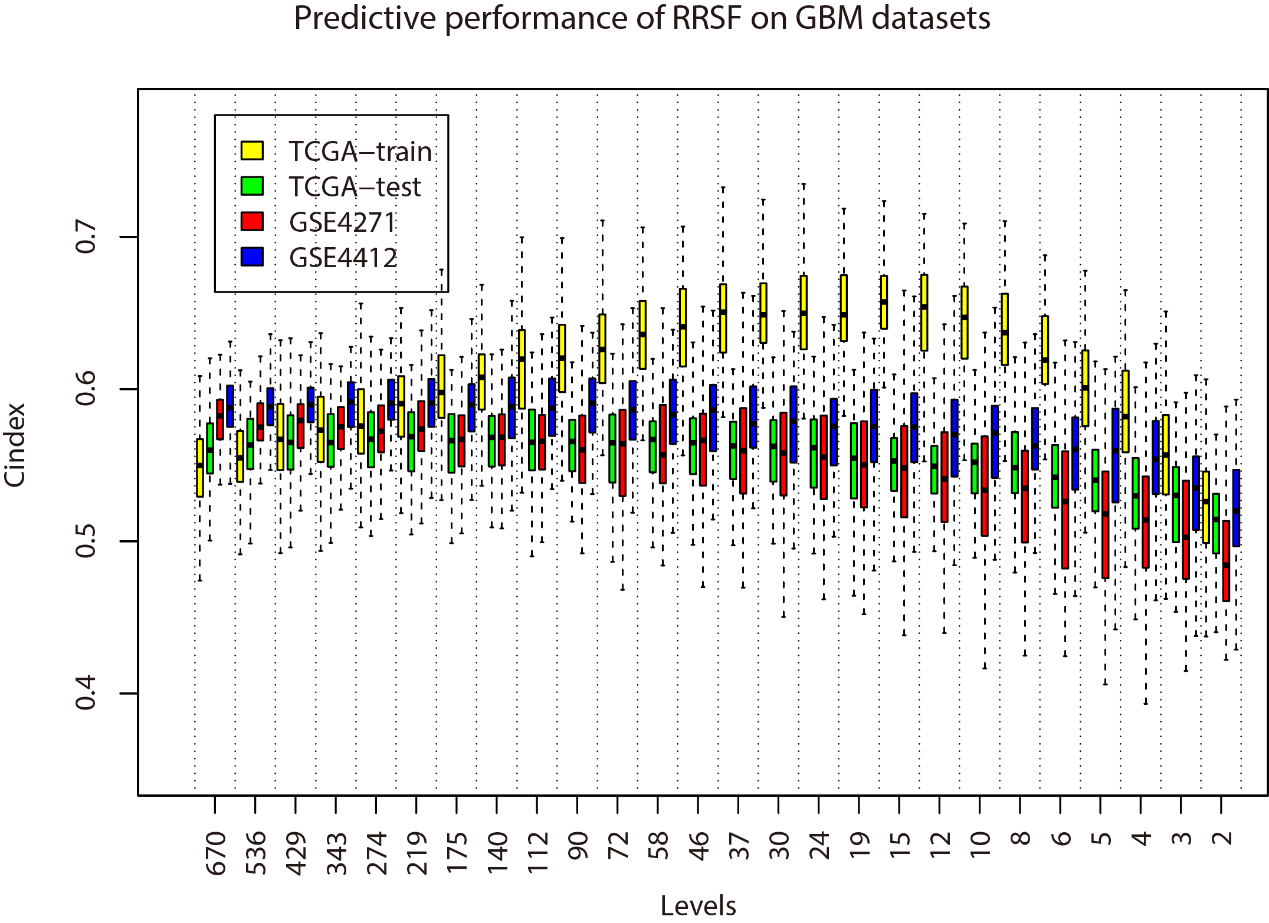


**Figure S21. Generalization performance of RRSF.** The dataset GBM-TCGA was randomly split into a training set (TCGA-train) and a test set (TCGA-test) 100 times. RRSF models trained on 100 TCGA-train datasets obtained similar predictive performance between TCGA-test and two independent datasets (GSE4412 and GSE4271).


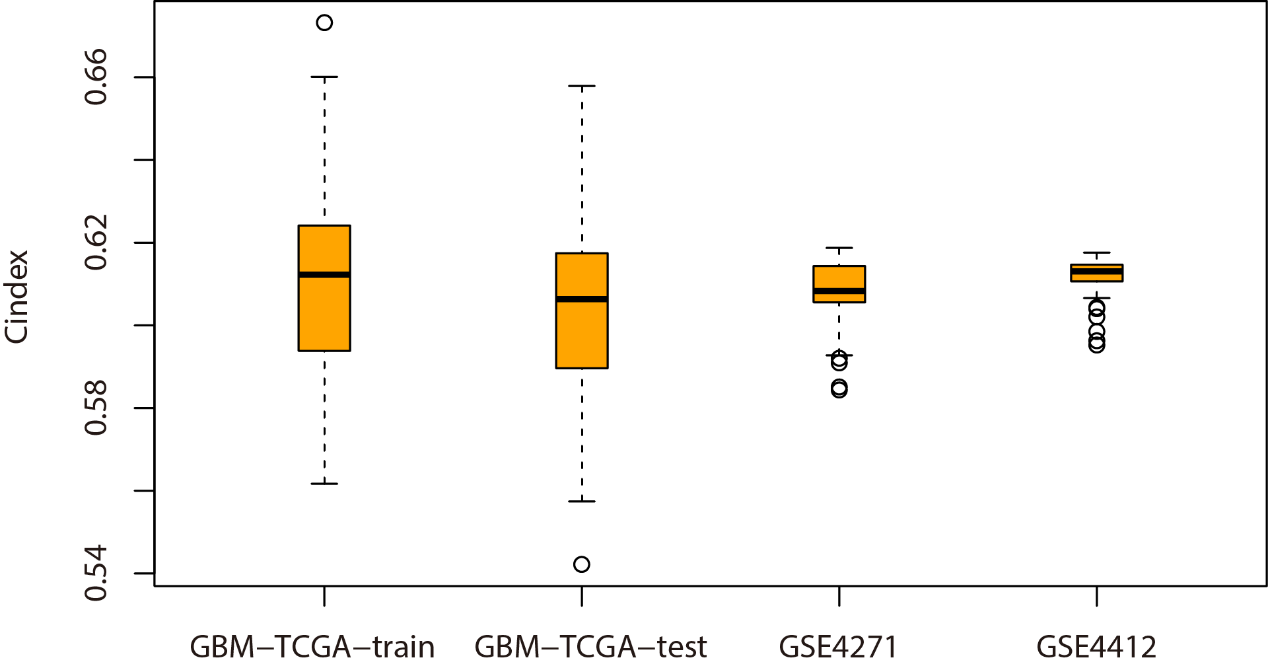


**Figure S22. Generalization performance of the two-gene signature.** The dataset GBM-TCGA was randomly split into a training set (TCGA-train) and a test set (TCGA-test) 100 times. The two-gene signature model trained on 100 TCGA-train datasets obtained similar predictive performance between TCGA-test and two independent datasets (GSE4412 and GSE4271).


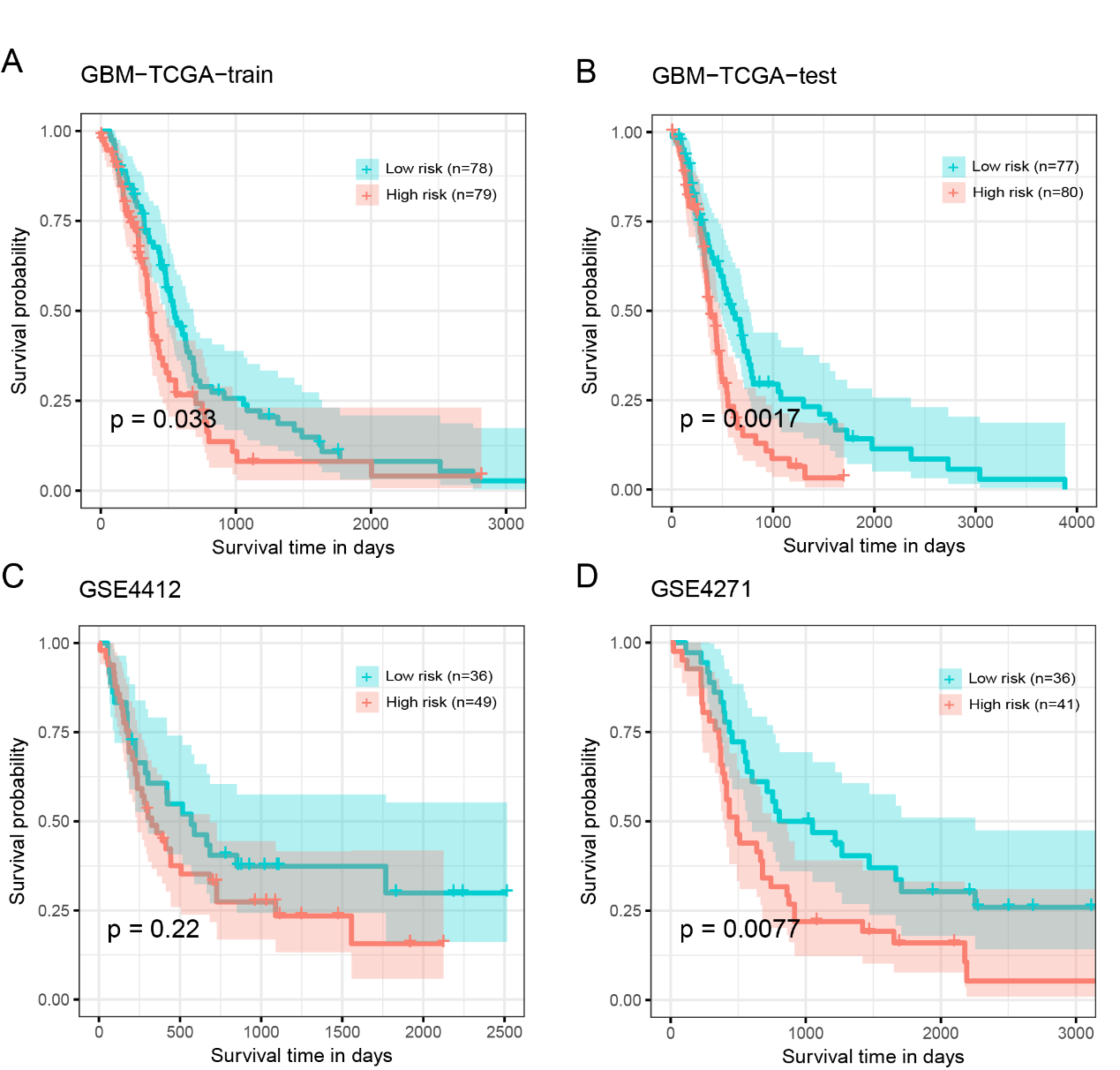


**Figure S23. Stratification of GBM patients using two genes selected by minimal depth.** The risk scores of patients were predicted by a Cox PH model trained on GBM-TCGA-train using two genes (*LAMB4* and *TANK*) selected by minimal depth. GBM patients were stratified into high- and low-risk groups using the mean of risk scores of patients in GBM-TCGA-train as the cutoff. P values were calculated by log-rank test.


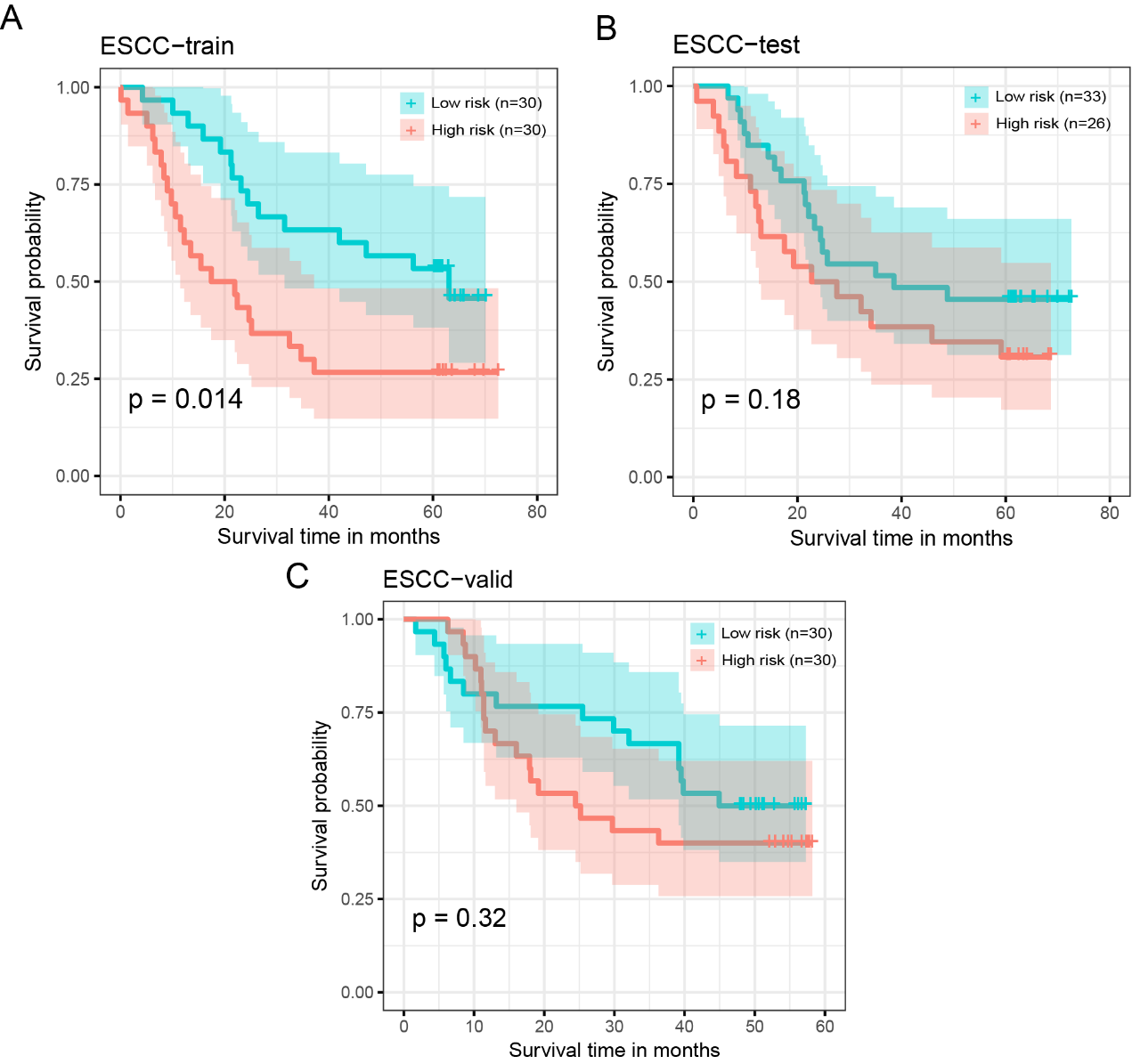


**Figure S24. Stratification of ESCC patients using three lncRNAs selected by minimal depth.** The risk scores of patients were predicted by a Cox PH model trained on ESCC-train using three lncRNAs (*DTX2P1-UPK3BP1-PMS2P11*, *AC114401.1* and *SNHG7*) selected by minimal depth. ESCC patients were stratified into high- and low-risk groups using the mean of risk scores of patients in ESCC-train as the cutoff. P values were calculated by log-rank test.


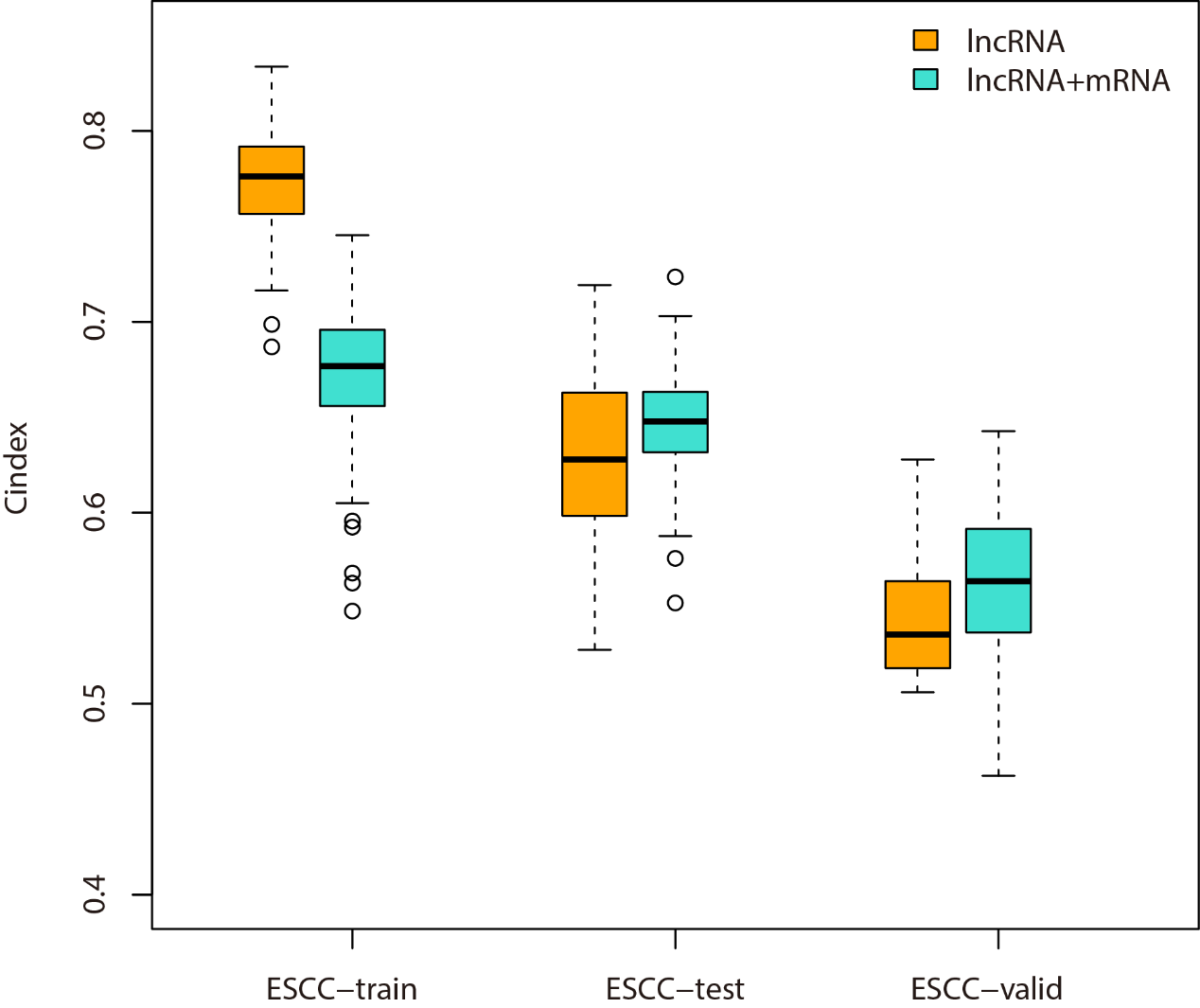


**Figure S25. Predictive performance of RRSF on ESCC datasets with lncRNA alone and with lncRNA+mRNA.** Shown are the C-indexes at 10-gene level. lncRNA, RRSF with lncRNAs as the initial feature set. lncRNA+mRNA, RRSF with both lncRNAs and mRNAs as the initial feature set.
